# Production of high-energy 6-Ah-level Li | |LiNi_0.83_Co_0.11_Mn_0.06_O_2_ multi-layer pouch cells via negative electrode protective layer coating strategy

**DOI:** 10.1038/s41467-023-39391-8

**Published:** 2023-06-19

**Authors:** Yangyang Feng, Yong Li, Jing Lin, Huyue Wu, Lei Zhu, Xiang Zhang, Linlin Zhang, Chuan-Fu Sun, Maoxiang Wu, Yaobing Wang

**Affiliations:** 1 State Key Laboratory of Structural Chemistry, Fujian Institute of Research on the Structure of Matter, Chinese Academy of Sciences, Fuzhou, 350002 P. R. China; 2 Fujian Key Laboratory of Nanomaterials, Fujian Institute of Research on the Structure of Matter, Chinese Academy of Sciences, Fuzhou, 350002 P. R. China; 3 State Key Laboratory of Space Power-Sources Technology, Shanghai Institute of Space Power-Sources, Shanghai, 200000 China; 4 College of Chemistry and Materials Science, Fujian Normal University, Fuzhou, 350007 China; 5 Fujian Science and Technology Innovation Laboratory for Optoelectronic Information of China, Fuzhou, 350108 P. R. China; 6 University of Chinese Academy of Sciences, Beijing, 100049 P. R. China

**Keywords:** Energy storage, Energy, Batteries, Materials for energy and catalysis, Electrochemistry

## Abstract

Stable lithium metal negative electrodes are desirable to produce high-energy batteries. However, when practical testing conditions are applied, lithium metal is unstable during battery cycling. Here, we propose poly(2-hydroxyethyl acrylate-co-sodium benzenesulfonate) (PHS) as negative electrode protective layer. The PHS contains soft poly (2-hydroxyethyl acrylate) and poly(sodium p-styrene sulfonate), which improve electrode flexibility, connection with the Cu current collector and transport of Li ions. Transmission electron cryomicroscopy measurements reveal that PHS induces the formation of a solid electrolyte interphase with a fluorinated rigid and crystalline internal structure. Furthermore, theoretical calculations suggest that the -SO_3_^-^ group of poly(sodium p-styrene sulfonate) promotes Li-ion motion towards interchain migration through cation-dipole interaction, thus, enabling uniform Li-ion diffusion. Electrochemical measurements of Li | |PHS-coated-Cu coin cells demonstrate an average Coulombic efficiency of 99.46% at 1 mA/cm^2^, 6 mAh/cm^2^ and 25 °C. Moreover, when the PHS-coated Li metal negative electrode is paired with a high-areal-capacity LiNi_0.83_Co_0.11_Mn_0.06_O_2_-based positive electrode in multi-layer pouch cell configuration, the battery delivers an initial capacity of 6.86 Ah (corresponding to a specific energy of 489.7 Wh/kg) and, a 91.1% discharge capacity retention after 150 cycles at 2.5 mA/cm^2^, 25 °C and 172 kPa.

## Abstract (web summary)

Stable lithium metal electrodes are needed to produce high-energy batteries. Here, authors reported poly(2-hydroxyethyl acrylate-co-sodium benzenesulfonate) as a lithium metal protective layer and the production of a 490 Wh/kg class Li | |LiNi_0.83_Co_0.11_Mn_0.06_O_2_ pouch cell.

## Introduction

High-specific-energy electrochemical storage is essential to pursue decarbonization and wireless society. Owing to the high theoretical capacity of 3860 mAh/g and the lowest reduction potential of −3.04 V (vs. standard hydrogen electrode), metallic lithium is the ideal negative electrode for high-specific-energy storage system^1,2^. For lithium metal batteries (LMBs), to achieve the specific energy beyond 400 Wh/kg, up to 500 Wh/kg, harsh conditions including high positive electrode loading ( > 4 mAh/cm^2^), thin lithium ( < 50 μm) and lean electrolyte ( < 2 g/Ah) are necessary (Supplementary Table 1)^3–6^. Notably, as calculated by various electrolyte amount with the different cathode loading (Supplementary Fig. 1), to realize the goal of 500 Wh/kg, the lean electrolyte of less than 1 g/Ah or the high-areal-capacity cathode exceeding 5 mAh/cm^2^ are required. Nevertheless, a severe reduction in the electrolyte amount can cause rapid decline in cell cycle life. Therefore, high cathode loading beyond 5 mAh/cm^2^ becomes particularly crucial to achieve the energy target. Unfortunately, paired with high-loading cathode, the high-areal-capacity lithium metal negative electrode is detrimentally affected by unfavorable Li metal deposition during cycling (e.g., dendrite formation) and volume change. 3D lithium host is considered as an efficient strategy to buffer the volume changes^7–9^, but at the cost of more Li and electrolyte consumption for solid electrolyte interphase (SEI) formation due to the large specific surface area, which unavoidably reduces coulombic efficiency and leads to capacity fading.

Therefore, the stable lithium-electrolyte interface plays a vital role to solve the above issues. Commonly, the in situ formation of native SEI is irregular and nonuniform, giving rise to uneven lithium deposition and continuous SEI degradation, which results in low Coulombic efficiency (CE), especially under high areal capacity^10,11^. Thus, attempting to design stable and functional protective layers to obtain uniform Li stripping/plating under high areal capacity is of interest. To date, some effective solutions have been proposed to obtain alternative protective layers, such as in situ formation of inorganic layers via electrolyte engineering^12–15^, and ex situ formation of artificial protective layers^6,16–19^ However, the in situ formed SEI layers hardly suppress large volume change under the high areal capacity of beyond 4 mAh/cm^2^ on cycling, causing SEI degradation and electrolyte consumption. Recently, various protective layers have been developed, such as inorganic materials, organic polymers, and inorganic-organic hybrid layers, to effectively strengthen the lithium-electrolyte interface and enhance the electrochemical performance^20–24^. Nevertheless, few can meet the satisfaction in pouch cells under practical conditions for high-specific-energy LMBs above 400 Wh/kg. Therefore, the rational design of artificial protective layers for stable high-areal-capacity lithium metal anode under realistic conditions is needed to produce high-specific-energy Li batteries.

Here, we design and develop a multi-functional artificial protective layer to regulate lithium deposition behavior by introducing poly(2-hydroxyethyl acrylate-co-sodium benzenesulfonate) (PHS) on Li metal electrode and Cu current collector surface via blade coating. On the one hand, the poly (2-hydroxyethyl acrylate) (PHEA) component of PHS can form bonds with Cu substrate via hydrogen-bond interaction as well as provide flexible segments for large volume change during cycling. On the other hand, the SO_3_^-^ groups in the poly(sodium p-styrene sulfonate) (PS) component of PHS can offer abundant Li^+^ adsorption sites and regulate Li^+^ diffusion, while Na^+^ can participate in the formation of NaF via ion exchange to contribute to hybrid fluorinated SEI. The rationally integrated PHS protective layer can induce the formation of high-crystalline inorganic inner SEI and facilitate uniform Li diffusion and flat Li deposition, leading to a high average CE of above 99% for Li | |PHS-coated-Cu coin cells (Li | |PHS-Cu) under high-areal-capacity of 8 mAh/cm^2^ at 1 mA/cm^2^, 25 °C. The density functional theory (DFT) calculations suggest that Li^+^ favors interchain migration from the surface of PHS to the current collector, beneficial for homogeneous lithium deposition at high areal capacity. When the PHS-coated Li metal negative electrode is paired with a high-areal-capacity (6 mAh/cm^2^) LiNi_0.83_Co_0.11_Mn_0.06_O_2_ (NCM83)-based positive electrode, in multi-layer pouch cell configuration, the battery delivers an initial capacity of 6.86 Ah (corresponding to an initial specific energy of 489.7 Wh/kg) and a 91.1% discharge capacity retention after 150 cycles at 2.5 mA/cm^2^, 25 °C, and 172 kPa.

## Results

**Fig. 1:**
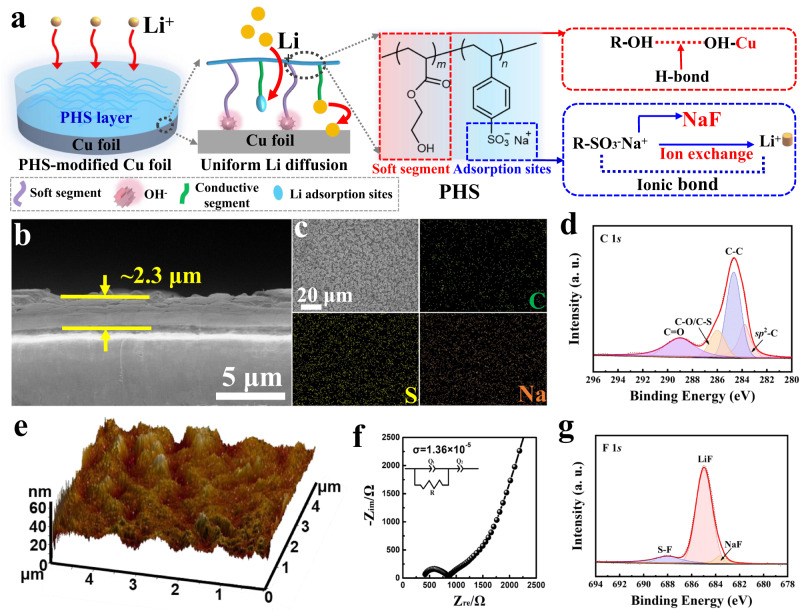
Physicochemical characterizations of the PHS-Cu electrode. **a** Schematic representation of the PHS-coated Cu electrode. **b** Cross-sectional SEM image of PHS-Cu. **c** The corresponding EDS mapping of PHS-Cu. **d** XPS spectrum of C 1 *s* for PHS-Cu. **e** 3D AFM topography image of PHS-Cu. **f** Complex impedance plot for the stainless steel|PHS|stainless steel symmetric cell to measure the PHS ionic conductivity, where the symbols indicate the raw data and lines refer to fitted data, with the Chi-Squared distribution (ChiSq) of 10^−4^. **g** XPS spectrum of F 1 *s* for PHS-Cu in Li | |PHS-Cu coin cells after plating 6 mAh/cm^2^ Li at 1 mA/cm^2^, 25 °C.

The PHS was synthesized via free radical polymerization with various PHEA/PS ratios (Supplementary Fig. 2). The soft PHEA in PHS can provide a flexible structure to buffer large volume change under high areal capacity and strong interaction with Cu due to the hydrogen-bond interaction^25^. Furthermore, the -SO^3-^ with large Li chemisorption energy (432.25 kJ/mol) in PS offers abundant Li^+^ adsorption sites to anchor Li^+^, which can effectively disperse Li^+^ and benefit from uniform diffusion along the PHS layers (Fig. 1a). Owing to the flexible property of PHEA (Supplementary Fig. 3) and ion conductivity of PS, the PHS with the PHEA/PS ratio of 1 exhibits better electrochemical performance. (Supplementary Fig. 4) Subsequently, the optimized PHS was bonded onto Cu foil through the blade-coating method (Fig. 1a), with an average thickness of 2.3 μm (Fig. 1b). The even distribution of C, S, and Na elements in Energy-dispersive X-ray spectroscopy (EDS) further confirms the uniformity (Fig. 1c). X-ray photoelectron spectroscopy (XPS) and Fourier transform infrared spectroscopy (FTIR) are conducted to verify the presence of PHS. Shown in Fig. 1d, the binding energies at 283.7, 284.6, 286.0, and 289.0 eV refer to the sp^2^ hybridized carbon, C-C, C-O, and C = O bonds respectively^26^. In the O 1 *s* and S 2*p* spectra, the -SO_3_^-^ is detected (Supplementary Fig. 5a, b). Combined with the Na 1 *s*, we can confirm that PHS is successfully coated on Cu foil via blade coating (Supplementary Fig. 5c). The C-H and -SO_3_^-^ stretching bands at ~3000 cm^−1^ and ~1182 cm^−1^ in FTIR also prove the PHS layers (Supplementary Fig. 6). In the 3D Atomic Force Microscope (AFM) morphology (Fig. 1e), PHS-Cu demonstrates the flat surface with a small fluctuation of ~40 nm. The Electrochemical impedance spectroscopy (EIS) measurement shows the high-ion-conductivity of 1.36 × 10^−5^ S/cm of PHS at 25 °C (Fig. 1f). After plating lithium on PHS-Cu via Li | |PHS-Cu asymmetric cells, LiF and NaF can be in situ formed during Li plating (Fig. 1g). The existence of -SO_3_Li implies that -SO_3_^-^ is lithiophilic and can absorb Li^+^ to transfer Li^+^ at the interface (Supplementary Fig. 7a), in accordance with the previous reported in literature^27,28^. Combined F 1 *s* with Li 1 *s* and Na 1 *s*, we can verify the formation of LiF and NaF at the interface (Supplementary Fig. 7b)^29^, indicating fluorinated hybrid SEI layer achieved by introducing PHS.

**Fig. 2:**
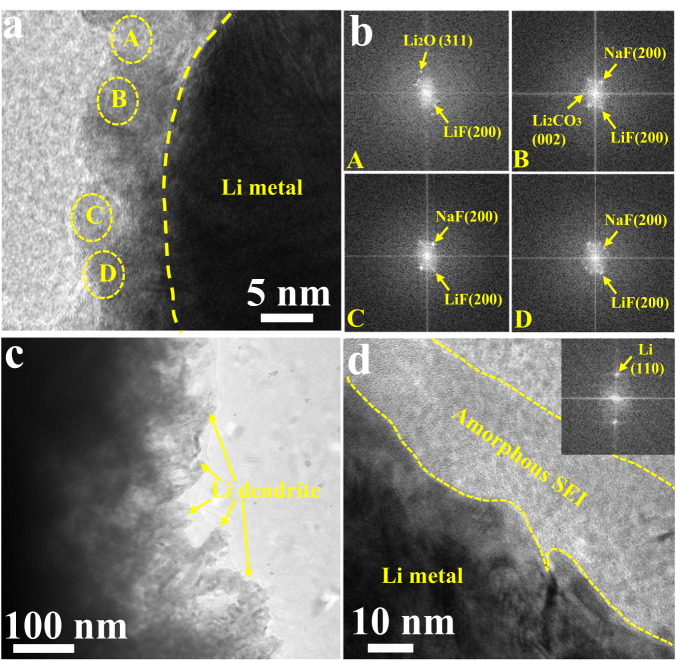
Structural, chemical and morphological characterization of the SEI structure. **a** Cryo-TEM image of the SEI layer formed on PHS-Cu in Li | |PHS-Cu coin cells after plating for 6 mAh/cm^2^ at 1 mA/cm^2^, 25  °C. **b** The corresponding local fast Fourier transform images of a). **c** Cryo-TEM image of the SEI layer formed on bare Cu in Li | |Cu coin cells after plating for 6 mAh/cm^2^ at 1 mA/cm^2^, 25  °C. **d** High-resolution TEM image of bare Cu. Inset of (**d**) is the corresponding local fast Fourier transform the image.

The structure and morphology of SEI under high areal capacity play vital roles in stable Li deposition to achieve high specific energy. Here, Cryo-TEM is conducted to investigate the SEI layer formed on the Cu current collector in Li | |PHS-Cu coin cells after plating high-areal-capacity of 6 mAh/cm^2^ lithium at 1 mA/cm^2^, 25 °C. As observed, the SEI layers formed under high areal capacity with/without modified PHS protective layer are completely different. From Fig. 2a and Supplementary Fig. 8, the SEI layer induced by PHS is highly crystalline with a hybrid layer including LiF, NaF, Li_2_O, and Li_2_CO_3_, achieving stable and high-ion-conductive inner layer^30^. The corresponding local Fourier transform images displayed in Fig. 2b clearly identify the lattice spacings of crystalline components in four parts, in which the lattice spacings of LiF, NaF, Li_2_O, Li_2_CO_3_ are 0.2 nm, 0.24 nm, 0.14 nm, 0.28 nm, respectively^31^. As reported, the fluorinated hybrid SEI possesses high mechanical strength and ionic conductivity, beneficial to relieving the large volume expansion during cycling^32^. Noteworthily, PHS-Cu presents a smooth lithium surface with a thin SEI layer of ~10 nm, suggesting stable and uniform Li deposition even under high areal capacity. However, bare Cu shows an entirely different SEI morphology, where numerous lithium depositions with dendrite morphology can be clearly detected under the high capacity of 6 mAh/cm^2^ (Fig. 2c). Increasing the TEM magnification, bare-Cu exhibits amorphous SEI with the thickness of ~30 nm (Fig. 2d).

**Fig. 3:**
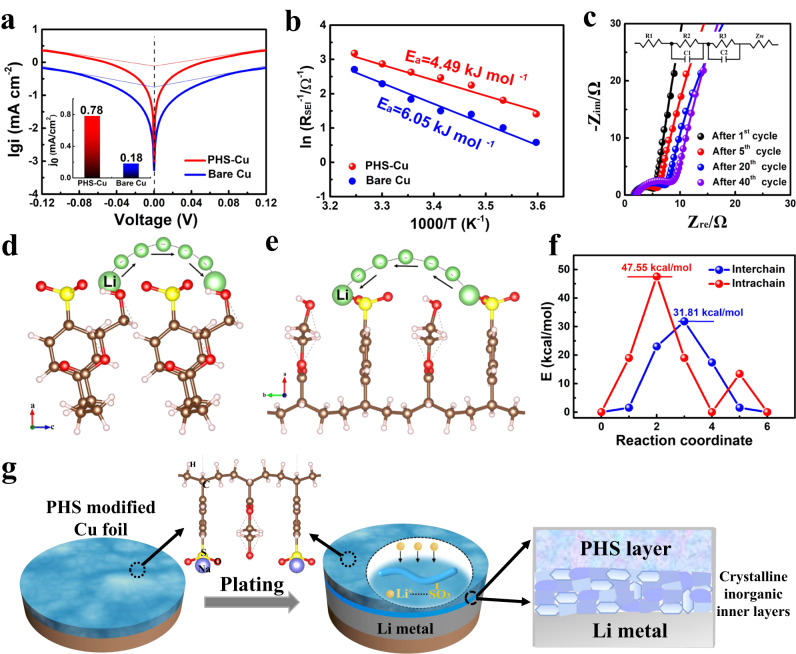
Study of the interfacial and kinetic properties of PHS-Cu. **a** Tafel plots of PHS-Cu/bare Cu in Li | |PHS-Cu and Li | |Cu coin cells tested at 25  °C. Inset is calculated exchange current density (jo). **b** Arrhenius plots of PHS-Cu/bare Cu and the calculated activation energy for Li^+^ diffusion. The temperatures calculated are in Kelvin. **c** Nyquist plots for Li | |PHS-Cu tested at different cycles under 25  °C, where the symbols indicate the raw data and lines refer to fitted data. Inset of (**c**) is the corresponding equivalent circuit. The model of Li^+^ migration behavior of (**d**) interchain and (**e**) intrachain. The green, yellow, red, brown, and pink spheres indicate Li, S, O, C, and H atoms, respectively. **f**) Theoretical calculation of Li^+^ migration energy. **g** Schematic illustrations of the Li plating behavior of PHS-Cu.

Besides the structure and components of SEI, the interfacial properties, and kinetics can also influence lithium deposition. The exchange current density (j_0_) and activation energy (E_a_) are also provided to evaluate the PHS protective layer. Figure 3a reveals the Tafel slopes and their corresponding j_0_ originated from intercept, from which PHS-Cu presents a j_0_ of 0.78 mA/cm^2^, much higher than that of bare Cu (0.18 mA/cm^2^), suggesting faster kinetics at the interface by modifying with PHS. Furthermore, the increased j_0_ indicates spherical nucleation and uniform deposition towards lithium during cycling^33^. The E_a_ obtained from temperature-dependent EIS measurements by linear fitting of ln(1/R) vs. 1/T is used to elucidate Li^+^ diffusion properties (Supplementary Fig. 9, Supplementary Tables 2–3). As observed in Fig. 3b, E_a_ of PHS-Cu is calculated as 4.49 kJ/mol, while that of bare C0.2 u is 6.05 kJ/mol, implying a smaller barrier and rapid Li-ion transport through the interface for PHS-Cu. The EIS measurements are also carried out to investigate the kinetic parameters at the electrolyte/electrode interfaces. From Fig. 3c and Supplementary Fig. 10, PHS-Cu demonstrates a small charge-transfer resistance (R_ct_) of 3.9 Ω, lower than that of bare Cu ( ~ 23.3 Ω), which indicates rapid charge transfer on PHS-Cu. It is noted that R_ct_ increases slowly from ~3.9 to ~7.3 Ω after 40 cycles ( ~ 480 h) under high areal capacity of 6 mAh/cm^2^ with 1 mA/cm^2^. On the contrary, bare Cu exhibits fast increase in R_ct_ from ~23.3 to ~ 41 Ω within only 10 cycles (Supplementary Fig. 11), which significantly revels the superior interfacial stability for PHS-Cu.

To further explore the Li-ion migration behavior through PHS, DFT calculations were provided. Figure 3d, e and Supplementary Fig. 12 demonstrates the atomic models of PHS with two different pathways of interchain and intrachain migration. In the initial state, Li^+^ favors to anchor on -SO_3_^-^ and then leaps along the polymer chain and via cation-dipole interaction (Supplementary Figs. 13–14). As calculated, Li-ion migration energy (E_m_) for interchain at any intermediate state is lower than 31.81 kcal/mol, much lower than the E_m_ for intrachain migration of 47.55 kcal/mol, indicating that the Li-ion prefers interchain migration (Fig. 3f). The calculation results confirm that the -SO_3_^-^ can adsorb/disperse Li^+^ and accelerate interchain migration from the surface to the current collector through the uniform PHS layers, which leads to smooth and flat lithium nucleation and growth. The above results confirm that multi-functional PHS layers with lithiophilic -SO_3_^-^ and Na^+^ can promote the formation of stable SEI layers and facilitate fast and uniform ion transfer. The schematic diagram is provided to better illustrate the Li deposition behavior (Fig. 3g).

**Fig. 4:**
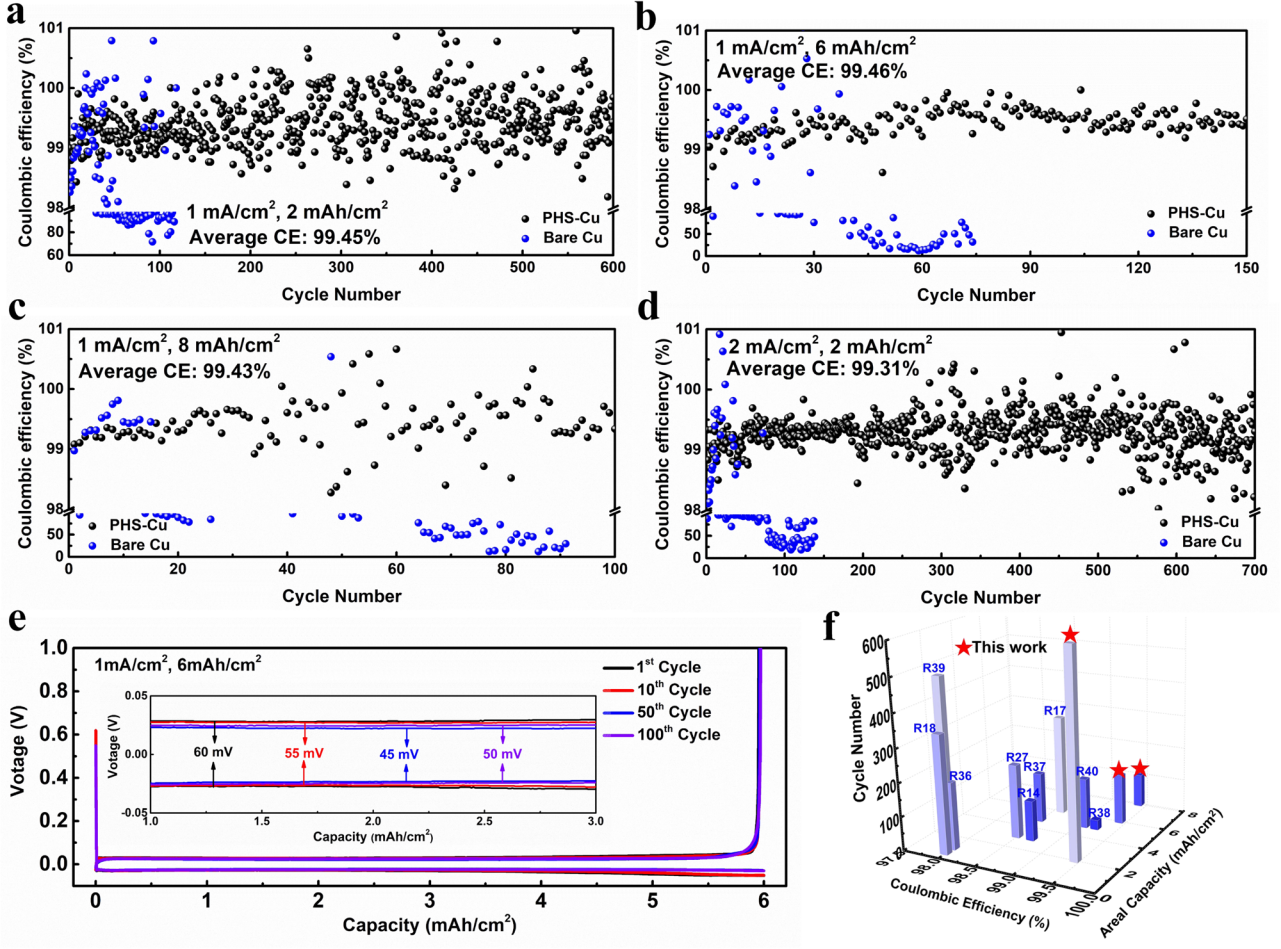
Electrochemical characterizations of Li | |PHS-Cu asymmetric cells. Cycling performance of Li | |PHS-Cu and Li | |Cu asymmetric cells with various capacity of (**a**) 2, (**b**) 6, (**c**) 8 mAh/cm^2^ at 1 mA/cm^2^ with the same amount of electrolyte of 70 uL tested at 25  °C. Notably, the cells are under the lean electrolyte of 11.67 μL/mAh and 8.75 μL/mAh at the capacity of 6 and 8 mAh/cm^2^, respectively. **d** Cycling performance at 2 mA/cm^2^ with 2 mAh/cm^2^ at 25  °C. **e** Voltage profiles of Li | |PHS-Cu asymmetric cell with 6 mAh/cm^2^ under 1 mA/cm^2^ at 25  °C. **f** Performance comparisons with recent works on Li | |Cu asymmetric cells, (R refers to reference in the main text, detailed in Supplementary Table 4).

To highlight the quality of PHS protect layer, a series of Li | |PHS-Cu cells are assembled to evaluate the electrochemical performance by using PHS-Cu/bare Cu as cathode and Li foil as the anode. Coulombic efficiency (CE) is a valid parameter to evaluate the cyclability of a Li metal electrode and it is defined as the ratio of stripping and plating capacity^34^. Here, we use CE to validate the effectiveness of PHS-Cu. Figure 4a–c show the plots of CE vs. cycle number under various areal capacities of 2, 6, and 8 mAh/cm^2^ at the same current density of 1 mA/cm^2^. As revealed in Fig. 4a, PHS-Cu exhibits high average CE of 99.45% with almost no CE decay for 600 cycles at the areal capacity of 2 mAh/cm^2^. On the contrary, bare Cu shows severe fluctuation in CE after only 50 cycles. When the areal capacity reaches 6 mAh/cm^2^, PHS-Cu delivers a high average CE of 99.46% for 150 cycles with negligible attenuation (Fig. 4b). Even when the areal capacity increases to 8 mAh/cm^2^, PHS-Cu remains stable at 100 cycles with the average CE of 99.43% (Fig. 4c). Notably, the Li | |PHS-Cu asymmetric cells ran under the lean electrolyte of 11.67 μL/mAh and 8.75 μL/mAh at the capacity of 6 and 8 mAh/cm^2^, respectively. In sharp contrast, the CE of bare Cu fluctuates in the early 20 cycles followed by a violent decrease and cell failure. To highlight the improvement of the PHS coating on Cu foil, a cycling test at a higher current density is also carried out. Observed in Fig. 4d, PHS-Cu demonstrates extraordinary stability with an average CE of 99.31% for 700 cycles at the high current density of 2 mA/cm^2^ with 2 mAh/cm^2^. However, for bare Cu, the CE drops rapidly within 100 cycles.

The corresponding voltage profiles are displayed in Fig. 4e under the capacity of 6 mAh/cm^2^ at various cycles, where Li | |PHS-Cu shows smooth and flat voltage plateaus with a low initial overpotential of ~60 mV followed by slightly decreasing to 50 mV at the beginning of 100 cycles, indicating the constant formation of stable SEI. Nevertheless, Li | |Cu presents fluctuated polarization curves with a larger overpotential of ~79 mV in the first cycle and rapidly increased overpotential of ~98 mV within 30 cycles (Supplementary Fig. 15). When the current density reaches 2 mA/cm^2^, Li | |PHS-Cu exhibits the overpotential of ~81 mV, while Li | |Cu shows much higher overpotential of ~146 mV, implying the fast ion diffusion after modified with PHS (Supplementary Fig. 16). The high CE and stability of the Li | |PHS-Cu are well-positioned compared to most of reported lithium metal anodes modified by various strategies (Fig. 4f, Supplementary Table 4)^14,17,18,35–40^. Furthermore, repeated lithium plating and stripping behaviors are also provided by Li | |Li symmetrical cells. As observed, the PHS-Li | |PHS-Li symmetric cell exhibits stability for 1000 h (250 cycles) under 2 mAh/cm^2^ at 1 mA/cm^2^ (Supplementary Fig. 17). In sharp contrast, for Li | |Li cell, the overpotential gradually increases upon cycling, followed by voltage fluctuation. The scanning electron microscopy (SEM) images (Supplementary Fig. 18) and XPS spectra (Supplementary Fig. 19) after cycling further confirm the stability of PHS layers.

**Fig. 5:**
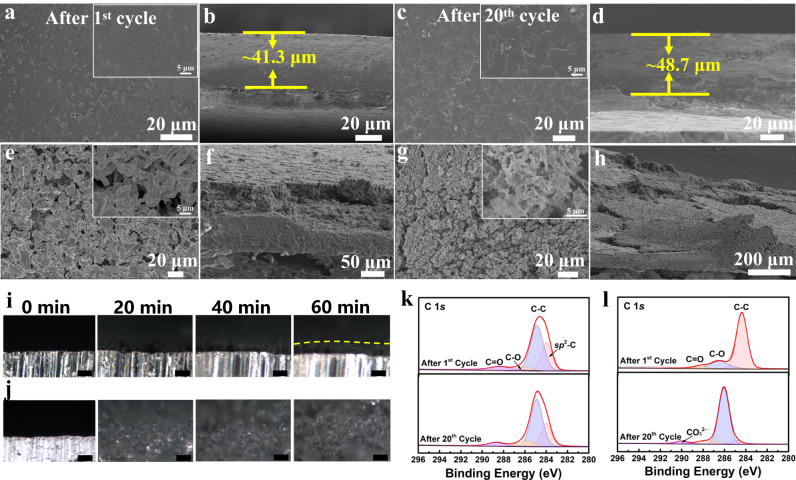
Physicochemical characterizations of the Li metal depositions on various Cu substrates. SEM images and Cross-sectional SEM images of (**a**–**d**) PHS-Cu in Li | |PHS-Cu and (**e**–**h**) Bare Cu in Li | |Cu at the plating process after (**a**, **b**), (**e**, **f**) 1^st^ and (**c**, **d**), (**g**, **h**) 20^th^ cycles at the current density of 1 mA/cm^2^ with areal capacity of 6 mAh/cm^2^ at 25  °C. In situ optical microscopy investigation of the interfaces between the electrolyte and electrode for (**i**) PHS-Cu in Li | |PHS-Cu and (**j**) bare Cu in Li | |Cu during lithium plating process (at 1 mA/cm^2^, 25  °C), the scale bar is 50 μm. XPS spectra of C 1 *s* for (**k**) PHS-Cu in Li | |PHS-Cu and (**l**) bare Cu in Li | |Cu after 1^st^ cycle and 20^th^ cycle with 6 mAh/cm^2^ at 1 mA/cm^2^, 25  °C.

To further study the Li plating behavior of PHS-Cu, SEM images after different cycles under 6 mAh/cm^2^ at 1 mA/cm^2^ are provided. As observed in Fig. 5a, b, PHS-Cu shows a smooth and dense surface with a thickness of ~41.3 μm at the plating process after 1^st^ cycle, slightly thicker than the theoretical value (~30 μm). Even after 20 cycles ( ~ 240 h), a uniform and flat Li morphology is obtained with a thickness of ~48.7 μm, indicating the uniform ion transfer and stable SEI attributed to PHS layers (Fig. 5c, d), which is also confirmed by the EDS mapping after 20 cycles (Supplementary Fig. 20). Unlike PHS-Cu, bare Cu shows irregular morphology after plating 6 mAh/cm^2^ (Fig. 5e). From the cross-sectional view, porous structures can be observed with a larger thickness of ~73 μm (Fig. 5f). After Li plating/stripping for 20 cycles, the needle-like lithium depositions (also including electronically disconnected lithium, known as “dead Li”) can be apparently observed with numerous cracks, (Fig. 5g, h), further implying that Li anode modified with PHS can facilitate spherical nucleation and dense Li deposition without dendrite growth. The improved stability of PHS-Cu is also verified by the corresponding SEM images after Li stripping (Supplementary Figs. 21 and 22). To gain more insight into the Li plating behavior, in situ optical microscopy (Supplementary Fig. 23) is carried out at 1 mA/cm^2^ via Li | |PHS-Cu and Li | |Cu asymmetric cells (Fig.  5i, j). As expected, PHS-Cu demonstrates uniform and dense morphology with a slight increase in thickness, while dendrite-like lithium grows rapidly on the bare Cu, extending to more than 100 μm in thickness.

In addition to SEM images, ex situ postmortem XPS electrode measurements are also carried out to prove the stability of the PHS coating. Figure 5k, l separately display the C 1 *s* spectra for PHS-Cu and bare Cu after 1 ^st^ and 20^th^ cycles. Owing to the PHS layer coating on the surface, C 1*s* spectra remain quite different from bare Cu. As observed, the spectra including sp^2^-C, C-C, C-O and C = O bonds are almost the same after 1^st^ and 20^th^ cycles for PHS-Cu. However, there appears a new peak at ~290 eV for bare Cu after 20 cycles, indexed to CO_3_^2−^ (Fig. 5l), indicating the decomposition of electrolyte upon cycling because of the decomposition of SEI layers^41^. Notably, the intensity of C-O bond sharply increases after 20 cycles, further implying the unstable SEI derived from dendrite growth, in accordance with SEM images. From the F 1 *s* spectra (Supplementary Fig. 24), we can detect that LiF and NaF peaks are on the surface of PHS-Cu with negligible changes after both 1^st^ and 20^th^ cycles, which further verifies the stable SEI during cycling. The S-F bond is derived from the lithium bis(fluorosulfonyl)imide (LiFSI) salt used as salt in the non-aqueous electrolyte solution^42^. In contrast, the relative intensity of LiF obviously decreases after 20 cycles, suggesting the unstable SEI for bare Cu.

**Fig. 6:**
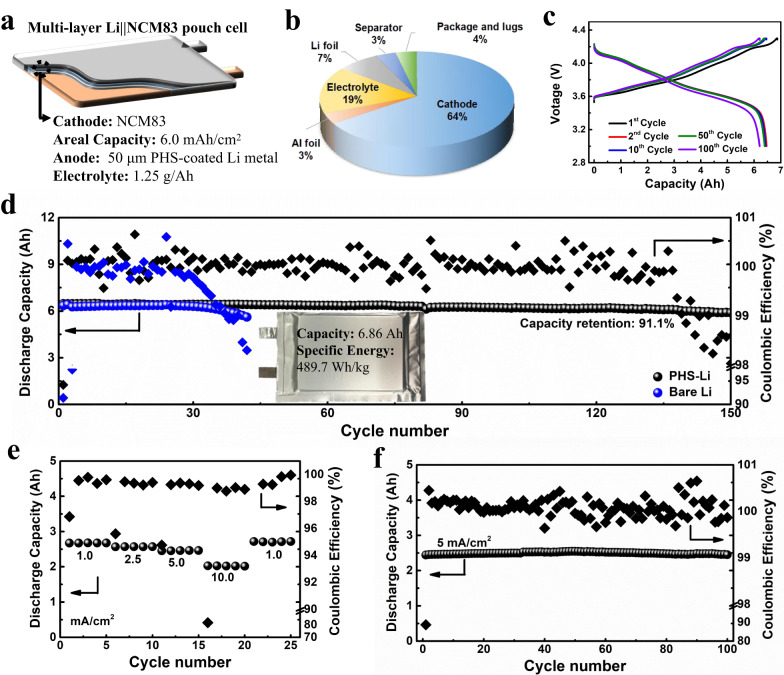
Electrochemical energy storage characterization of the Li | |NCM83 pouch cells. **a** Schematic diagram of a pouch cell under practical conditions with a thin Li anode, lean electrolytes, and high-loading cathode, where blue, white and orange layers refer to cathode, separator, and anode, respectively. **b** The pie chart of the mass distribution of all cell components in a pouch cell. The specific energy in this work is calculated by the whole mass in the pouch cell including the package and lugs. **c** The voltage profiles of PHS-Li | |NCM83 pouch cell at different cycles. **d** Cycling performance of PHS-Li | |NCM83 and Li | |NCM83 pouch cells at 0.5 mA/cm^2^ for charging and 2.5 mA/cm^2^ for discharging at 25  °C. Inset is the optical photograph of the pouch cell. **e** Rate performance of PHS-Li | |NCM83 pouch cell at the constant charge current of 0.5 mA/cm^2^ and various discharge currents from 1 to 10 mA/cm^2^ at 25  °C. **f** Cycling performance at 0.5 mA/cm^2^ for charging and 5 mA/cm^2^ for discharging at 25  °C.

Benefiting from the electrochemical behavior of PHS-Cu, we applied harsh practical conditions to assemble pouch cell to evaluate the PHS coating potential for high-specific-energy LMBs (Fig. 6a). The pouch cell is fabricated by coupling high-areal-capacity LiNi_0.83_Co_0.11_Mn_0.06_O_2_ (NCM83) cathode (6 mAh/cm^2^) and PHS-coated Li metal negative electrode (50 μm) with lean electrolyte ( ~ 1.25 g/Ah). In order to tolerate the high-voltage of NCM83, carbonate-based electrolyte (0.6 M LiBF_4_ and 0.6 M LiBOB in 2:1 DEC/FEC) is applied instead of ether-based electrolyte. Here, the as-assembled pouch cell with high capacity of 6.86 Ah (calculated by discharge capacity in the battery formation process, Supplementary Fig. 25) delivers high specific energy of 489.7 Wh/kg, calculated by the total mass of all parts including cathode, anode, current collectors, electrolyte, separator and package (Fig. 6b, Supplementary Table 5). Figure 6c shows the voltage profiles of PHS-Li | |NCM83 pouch cell, where the pouch cell reveals stable voltage plateaus with low voltage polarization even after 100 cycles. The long-term cyclic stability is presented in Fig. 6d, where the cell is cycled for 150 cycles at 2.5 mA/cm^2^ with a high capacity retention of 91.1%. In contrast, Li | |NCM83 pouch cell just exhibits stability for the initial 40 cycles followed by sharp attenuation. Notably, the Coulombic efficiencies remain above 99.8% during long-time cycling, with slight decrease in the last few cycles.

To investigate the degradation mechanism, the optical photographs of PHS-Li | |NCM83 and Li | |NCM83 pouch cells after cycling until the Coulombic efficiency lower than 98% are provided (after 150 cycles for PHS-Li | |NCM83 and 42 cycles for Li | |NCM83). As observed, the NCM83 cathode has almost no change and can maintain stability over long cycles (Supplementary Fig. 26a, b), indicating that the main degradation should come from the anode side, as already described in the literature^4^. In Supplementary Fig. 26c, the unreacted Li can be detected after cycling, suggesting that the electrolyte depletion is the main factor of efficiency decrease. For PHS-Li | |NCM83 cell, the primary cause should be electrolyte depletion. From Supplementary Fig. 27a, b, we find that a small amount of electrolyte (0.5-1 g/Ah) is left in the cell, while the Li is almost consumed (Supplementary Fig. 27c), suggesting that Li consumption and the dead Li formation should cause the efficiency decrease for Li | |NCM83 pouch cell. The above results indicate that PHS layer can stable the Li/electrolyte interface, retard the consumption of lithium and electrolyte, which further implies that uniform lithium diffusion and deposition are crucial to prolong the cycle life under high areal capacity. In addition, the influence of electrolyte amount on cycle life is also investigated to confirm the importance of uniform Li deposition. Li | |NCM83 pouch cells with various electrolyte amounts are assembled with different electrolyte amounts. In Supplementary Fig. 28, the lifetime of the pouch cells prolongs from 37 to 64, 86, 99, and 108 cycles with the electrolyte increases from 1.05 g/Ah to 1.26, 1.53, 1.89, and 2.2 g/Ah, respectively (Supplementary Table 6). Notably, the cycle life cannot be significantly prolonged as the electrolyte amount increases when the electrolyte retention is more than 1.5 g/Ah, suggesting that the electrolyte amount may not be a decisive factor affecting the cycle life under high areal capacity and low N/P ratio, when the electrolyte exceeds a certain amount.

To highlight the suitability of PHS coating for practical applications, the rate performance is also evaluated. Figure 6e and Supplementary Fig. 29 show the rate capability tests of PHS-Li | |NCM83 pouch cell, in which PHS-Li | |NCM83 exhibits good rate capabilities from 1 to 10 mA/cm^2^ at the charging current of 0.5 mA/cm^2^, delivering a high capacity of 2.1 Ah at 10 mA/cm^2^ as well as high CE. In addition, the cell exhibits almost no capacity attenuation ( ~ 100% capacity retention) for 100 cycles at 5 mA/cm^2^ (Fig. 6f and Supplementary Table 7), further confirming the potential of the PHS coating strategy for practical battery applications. Besides, the rate performance at a higher charging current of 1 mA/cm^2^ is also conducted to verify its practicability (Supplementary Fig. 30).

In summary, we report a stable high-areal-capacity lithium metal anode by homogeneously introducing a PHS polymer protective layer to produce a 490 Wh/kg class LMBs. Benefiting from the soft segments of PHEA with the strongly interaction with Cu substrate, PHS protective layer can not only evenly and smoothly coat on the Cu foil, but also buffer huge volume changes during cycling under high areal capacity. The PS in PHS can enrich Li^+^ adsorption sites, disperse Li^+^, and accelerate Li^+^ towards interchain migration along -SO_3_^-^ through cation-dipole interaction across the interface and access to the current collector, endowing fast Li^+^ transfer rate and uniform lithium nucleation and growth. Therefore, the assembled Li | |PHS-Cu asymmetric cells exhibit an average CE of 99.43% for 100 cycles even under a high areal capacity of 8 mAh/cm^2^ with a lean electrolyte of 8.75 μL/mAh. When the PHS-coated Li metal negative electrode is paired with a high-areal-capacity (6 mAh/cm^2^) NCM83-based positive electrode, in a multi-layer pouch cell configuration, the battery delivers an initial capacity of 6.86 Ah (corresponding to the initial specific energy of 489.7 Wh/kg) and a 91.1% discharge capacity retention after 150 cycles at 2.5 mA/cm^2^, 25 °C, and 172 kPa.

## Methods

### Synthesis of Poly(2-hydroxyethyl acrylate-co-sodium benzenesulfonate) (PHS)

The PHS was synthesized via the free radical polymerization of 2-hydroxyethyl acrylate (HEA, 97%, Aladdin) and sodium p-styrene sulfonate (S, >90%, Aldrich). 1.4 g HEA, 1.4 g S, 10 mg ammonium persulfate (APS, ≥98%, Aldrich), and 6 g deionized water were transferred into a round bottom flask (25 mL) equipped with a magnetic stir bar. Then the mixture was degassed at 25  °C for 10 min under vacuum. After three times degassing, the mixture was heated for 1 h at 65 °C. The resulting product was purified by dialyzing (7 kDA) in water to obtain PHS. The other weight ratios of HEA and S were also controlled at 3:7, 4:6, 6:4, and 7:3, and synthesized for comparison with the same procedure.

### Preparation of the electrodes for Li metal coin cell testing

For PHS-Cu anode, at first, the as-synthesized PHS was added in tetrahydrofuran (THF) to form a homogeneous solution (0.05 wt%). The Cu substrates were cleaned and washed under acetone for 20 min and fast rinsed in water for 5 times and finally dried in a vacuum oven at 60  °C. Then, adding the PHS solution onto the surface of Cu foil through blade coating by adjustable coater on an automatic coating machine. After that, the PHS-coated Cu foil was dried at 60  °C for 2 h to remove the solvent and punched into pieces with a diameter of 16 mm by a coin cell punching machine. The PHS modified Li foil is prepared under the same conditions as PHS-Cu.

Li | |PHS-Cu and Li | |Cu cells were assembled in Ar-filled glove box (H_2_O, O_2_ < 0.1 ppm) by using CR2032 coin cells. The PHS-Cu and Cu foil ( > 99.9%, 8 μm, Chang Chun Petrochemical Co., Ltd.) are used as working electrodes and Li foil ( ~ 400 μm) are used as counter electrode. All the coin cells are under the same electrolyte of 4 M lithium bis(fluorosulfonyl)imide (LiFSI) in Methoxymethane (DME) (the water content is ~12 ppm). Then, the as-assembled coin cells were tested with various capacities from 2 mAh/cm^2^ to 6 and 8 mAh/cm^2^ at 1 or 2 mA/cm^2^ with the same electrolyte amount of 70 μL. More than six cells have been tested for each electrochemical experiment. All the coin cells were tested under 25  °C ± 0.5  °C in a constant temperature room.

### Preparation of the electrodes for Li metal multi-layer pouch cell testing

The assembly and test of pouch cells were conducted in State Key Laboratory of Space Power-Sources Technology, Shanghai Institute of Space Power-Sources. The cathode was obtained by mixing LiNi_0.83_Co_0.11_Mn_0.06_O_2_ (NCM83), polyvinylidene difluoride (PVDF), Super P, and vapor-grown carbon fiber (VGCF) with a weight ratio of 95.5:2.2:1.8:0.5 in N-methyl-2-pyrrolidone (NMP). The cathode slurry was coated on both sides of Al foil ( > 99%, 12 μm, Hebei Xingheng New Materials Technology Co., Ltd.). And then the electrode film was calendared at 5 r/min under 20 T by hydraulic counter-roll machine, punched into rectangular pieces of 5.6 × 8.0 cm, and dried in a vacuum oven (80 °C, 24 h). The prepared cathode has a single-sided areal mass loading of 30 mg/cm^2^ with a thickness of 172 μm (including Al foil) and a porosity of ~35%. The anode was prepared by coating PHS layer on the double-side electrode surface of Li foil (99.95%, 100 μm × 82 mm, China Energy Lithium) and Cu foil ( > 99.9%, 8 μm, Chang Chun Petrochemical Co., Ltd.) Firstly, peel off the plastic film originally wrapped on the Li foil, then drop a small amount of PHS solution on the Li foil and cover the plastic film on Li foil. After the formation of uniform PHS coating on Li foil, peel off the plastic film. The PHS-Cu was prepared via the same process.

The pouch cells were assembled in a dry room with a dew point below −55  °C at a temperature of 20  °C by using NCM83 as cathodes and PHS-Li/PHS-Cu as anodes and 0.6 M lithium tetrafluoroborate (LiBF_4_) and 0.6 M lithium bis(oxalate)borate (LiBOB) in 2:1 diethyl carbonate/ fluoroethylene carbonate (DEC/FEC) as electrolyte with the commercial separator (25 μm. Celgard 2325, Tianjing KPRT Co., Ltd). The pouch cells were assembled with a stacked process. Each pouch cell contains 13 layers of double-side cathode and 14 layers of double-side anode, separated by a separator, packed by Al-plastic film (73 μm, Dai Nippon Printing Co., Ltd). The detailed parameter is presented in Supplementary Table 5. The pouch cell was tested at 0.5 mA/cm^2^ for charging and 2.5 mA/cm^2^ and 5 mA/cm^2^ for discharging, during which the pouch cell was sandwiched in the stainless-steel clamping device with an applied external pressure of ~172 kPa (Supplementary Fig. 31). All the pouch cells were tested under 25  °C ± 0.5  °C in high-low temperature test chamber. More than three cells have been tested for each electrochemical experiment.

### Ex situ physicochemical characterization

The FTIR spectrum of PHS-Cu was tested by Fourier transform infrared spectroscopy (FTIR, Bruker) at 25  °C in the range of 500 to 4000 cm^−1^. The Stress-strain curve of PHEA was obtained by Tensile testing machine (PN-TT300) at the stretching speed of 5 mm/min. The topography image is obtained by Atomic force microscope (AFM, NT-MDT). The morphologies and structural analysis of electrodes at different conditions before and after cycling were measured by field-emission scanning electron microscope (SEM) coupled with an EDS analyzer (Hitachi, SU8010, 5 kV) and X-ray photoelectron spectroscopy (Thermo FisherScientific, Al (Ka)). After cycling, the coin cells were disassembled and the anode was washed by DME for several times to remove the lithium salts on the surface of the anode in the Ar-filled glove box (H_2_O, O_2_ < 0.1 ppm) before the test. The samples were sealed hermetically inside an air-tight can and transferred to the instrument chamber in air.

The Cryo-TEM (Titan Krios G3i 300 kV) is used to investigate the SEI structure and component. Firstly, we assembled Li | |PHS-Cu coin cells. After plating 6 mAh/cm^2^ lithium on PHS-Cu at 1 mA/cm^2^, the cell was disassembled and the anode was washed by DME for several times to remove the lithium salts on the surface of anode in the Ar-filled glove box. Then scrape the lithium from PHS-Cu electrode and dissolve it in DME. After that, we drop the solution on the TEM grids and dry it in Ar-filled glove box (H_2_O, O_2_ < 0.1 ppm). The grid sample was then loaded on the cryo-TEM holder and inserted into the microscope without exposure to air.

### In situ optical microscopy

In situ optical microscopy was performed via a home-made side-by-side cell (Li | |PHS-Cu and Li | |Cu) (The schematic diagram is provided in Supplementary Fig. 23). The home-made cell was assembled at 25  °C in an argon-filled glove box with water and oxygen contentless than 0.1 ppm. The in situ optical diagrams are obtained by the cross-sectional view of PHS-Cu during the plating process at 1 mA/cm^2^ under the optical microscope from Olympus.

### Electrochemical measurements

The galvanostatic charge/discharge profiles were tested by Neware battery testers (Shenzhen, China). The AC impedance spectroscopy, the activation energy of cells, and the exchange current density were conducted in CHI660 electrochemical workstation. AC impedance was performed between 0.1 Hz to 10^5^ Hz with 12 points per decade of frequency using the voltage amplitude of 10 mV. All the EIS are tested at open-circuit voltage. All the Nyquist plots are fitted by the equivalent circuit, where the Chi-Squared distribution (ChiSq) is between 1.6 × 10^−4^ and 6.9 × 10^−3^.

Activation energy measurements were tested by using Li | |PHS-Cu and Li | |Cu cells through EIS characterization in an incubator under different temperatures from 5 to 35  °C with 5  °C separation, where the variation of temperature was controlled to be less than 0.1 °C. Activation energy value was calculated through the Arrhenius equation:

1 $$T/{R}_{{SEI}}=A{{{{{\rm{exp }}}}}}(-{E}_{a}/{RT})$$

Where *E*_*a*_ is the activation energy, *T* is the absolute temperature, *R* is the gas constant, *R*_*SEI*_ (obtained by fitting the EIS raw data of the high-frequency semicircle) is the interfacial Li^+^ transfer resistance, and A is the pre-exponential factor.

Ionic conductivity of PHS was measured by using AC impedance spectroscopy with a perturbation voltage of 10 mV in the frequency range from 10^5^ Hz to 0.1 Hz. Symmetrical stainless-steel (304 stainless steel, 16 mm in diameter, 1 mm in thick, Xinghua Muxin New Energy Materials Co. Ltd) SS | PHS | SS cells were assembled to test the resistance value under 25 °C ± 0.5  °C. The ionic conductivity (σ, in S/cm) was calculated by the following equation:

2 $$\sigma=L/{R}_{b}S$$

where L (cm) is the thickness of PHS membrane, *R*_*b*_ (obtained by fitting the EIS raw data of the high-frequency semicircle) is the bulk resistance of the PHS membrane, and S (cm^2^) is the electrode area.

### Calculation of specific energy

The specific energy of the Li | |NCM83 pouch cell in the manuscript is calculated by the following equation:

3 $$E={C}_{{cell}}\times {V}_{{cell}}/{W}_{{cell}}$$

Where *C*_*cell*_ is the cell capacity (Ah), *V*_*cell*_ is the average cell discharge voltage and *W*_*cell*_ is the total mass of the cell (kg) including a positive electrode, a negative electrode, electrolyte, separator, package, and lugs. The specific values of the cell are provided in Supplementary Table 5.

## Supplementary information

Supplementary Information

## Data Availability

The data generated in this study are provided in the Source Data file. Source data are provided with this paper.

## Source data

Source data
