# Supplementary material for: Production of high-energy 6-Ah-level Li | |LiNi0.83Co0.11Mn0.06O2 multi-layer pouch cells via negative electrode protective layer coating strategy
Source: Nat Commun. 2023 Jun 19;14:3639. doi: 10.1038/s41467-023-39391-8 (PMC10279762; doi:10.1038/s41467-023-39391-8)
Supplement: Supplementary file 1 — Supplementary Information [file 41467_2023_39391_MOESM1_ESM.pdf]

Production of high-energy 6-Ah-level Li||$LiNi_{0.83}Co_{0.11}Mn_{0.06}O_2$ multi-layer pouch cells via negative electrode protective layer coating strategy

Yangyang Feng[1,2], *Yong Li*[3]*, *Jing Lin*[1,2], *Huyue Wu*[1,2,4], *Lei Zhu*[1,2], *Xiang Zhang*[1,2], *Linlin Zhang*[1,2], *Chuan-Fu Sun*[1,2], *Maoxiang Wu*[1,2], *Yaobing Wang*[1,2,5,6]*

[1]State Key Laboratory of Structural Chemistry, Fujian Institute of Research on the Structure of Matter, Chinese Academy of Sciences, Fuzhou, 350002, P. R. China

[2]Fujian Key Laboratory of Nanomaterials, Fujian Institute of Research on the Structure of Matter, Chinese Academy of Sciences, Fuzhou, 350002, P. R. China.

[3]State Key Laboratory of Space Power-Sources Technology, Shanghai Institute of Space Power-Sources, Shanghai 200000, China.

[4]College of Chemistry and Materials Science, Fujian Normal University, Fuzhou 350007, China.

[5]Fujian Science and Technology Innovation Laboratory for Optoelectronic Information of China; Fuzhou 350108, Fujian, P. R. China.

[6]University of Chinese Academy of Sciences; Beijing 100049, P. R. China.

These authors jointly supervised this work: Yong Li, Yaobing Wang

*Correspondence: ydx1112@126.com (Yong Li); wangyb@fjirsm.ac.cn (Yaobing Wang)

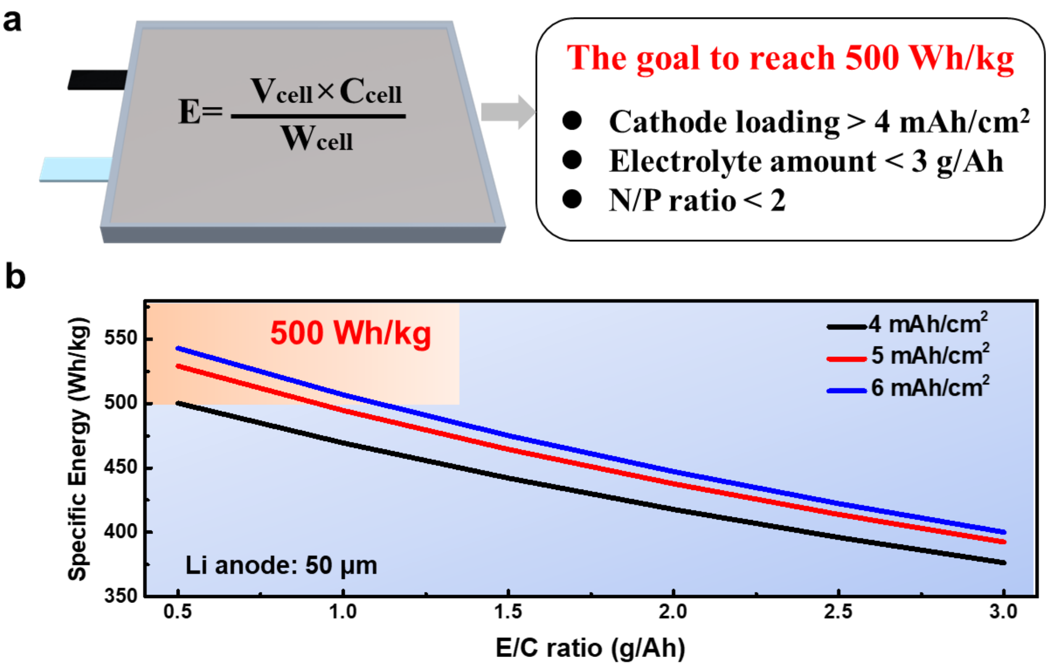

Supplementary Figure 1. The pivotal paraments to achieve 500 Wh/kg. a) Schematic diagram of pouch cell under practical conditions with thin Li anode, lean electrolytes, and high-loading cathode. Inset is the equation of specific energy, where C_{cell} is the cell capacity (Ah), V_{cell} is the average cell discharge voltage of the cell and W_{cell} is the total mass of the cell (kg) including positive electrode, negative electrode, electrolyte, separator, package and lugs. b) The specific energy calculated by different electrolyte amount with the cathode loading of 4, 5 ,6 mAh/cm^2. The other cell parameters are kept the same as that in Supplementary Table 3.

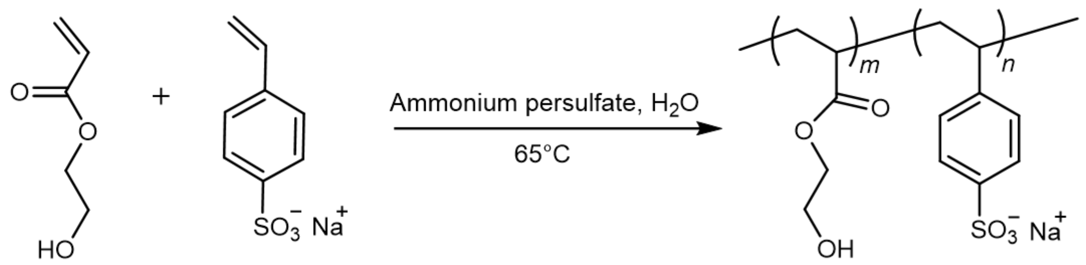

Supplementary Figure 2. Schematic diagram of the fabrication of PHS via a free radical polymerization. The ratios of m and n are 3:7, 4:6, 5:5, 6:4 and 7:3.

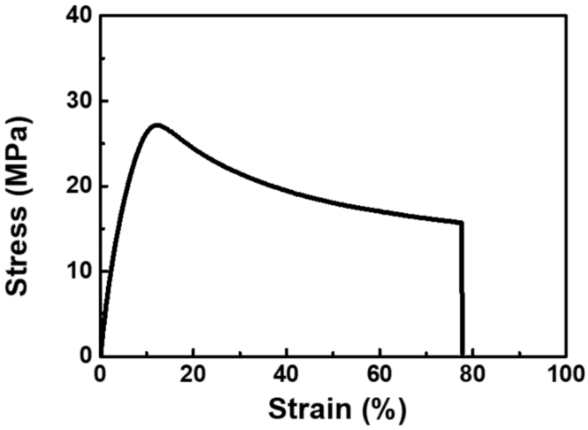

Supplementary Figure 3. Stress-strain curve of PHEA. The PHEA film achieves ~77% strain before fracture.

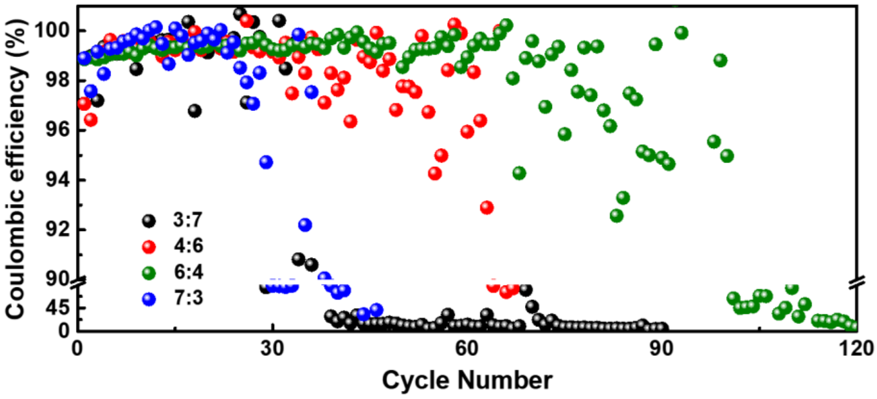

Supplementary Figure 4. Cycling performance of Li||PHS-Cu asymmetric cells with various PHEA/PS ratio of 3:7, 4:6, 6:4 and 7:3 under 6 mAh/cm^2 at 1 mA/cm^2 with the same amount of electrolyte of 70 uL at 25 ^{o}C. These results show that the electrochemical performance is better when the PHEA/PS ratio close to 1, indicating the equal importance of PHEA and PS as one can provide soft property and one can enhance the conductivity.

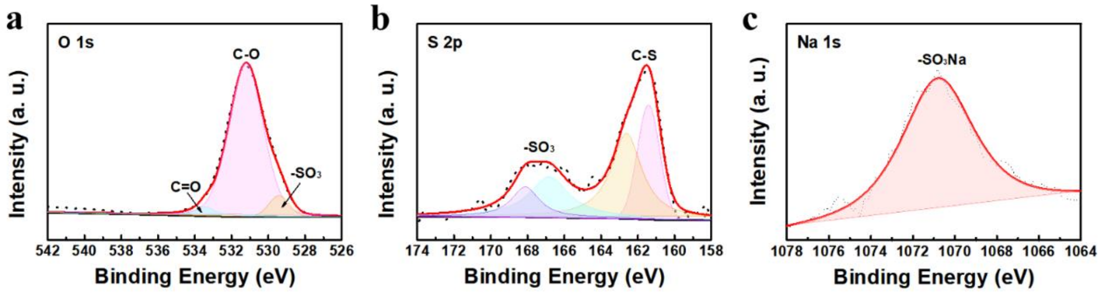

Supplementary Figure 5. XPS spectra of a) O 1*s*, b) S 2*p* and c) Na 1*s* for PHS-Cu before cell cycling.

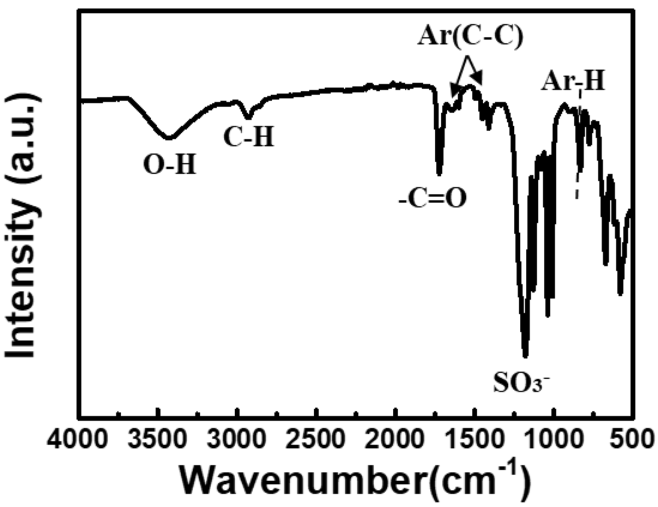

Supplementary Figure 6. FTIR spectrum of PHS-Cu before cell cycling.

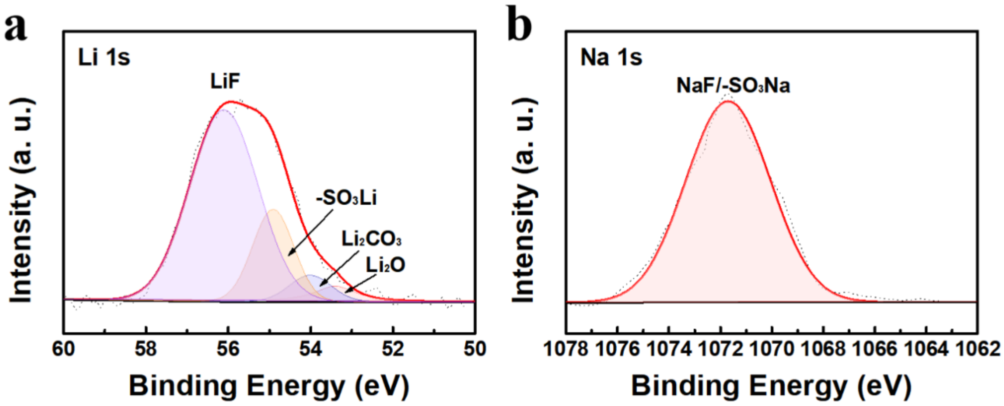

Supplementary Figure 7. XPS spectra of a) Li 1*s* and b) Na 1*s* for PHS-Cu in Li||PHS-Cu after 1st cycle under 6 mAh/cm^2 at 1 mA/cm^2, 25 ^oC. In the Li 1*s* spectrum, the binding energies at 53.2, 54, 54.9, 56.2 eV are assigned to Li_2O, Li_2CO_3, -SO_3Li and LiF, respectively. In the Na 1*s* spectrum, the peak at 1071.7 eV corroborates the existence of NaF and -SO_3Na.

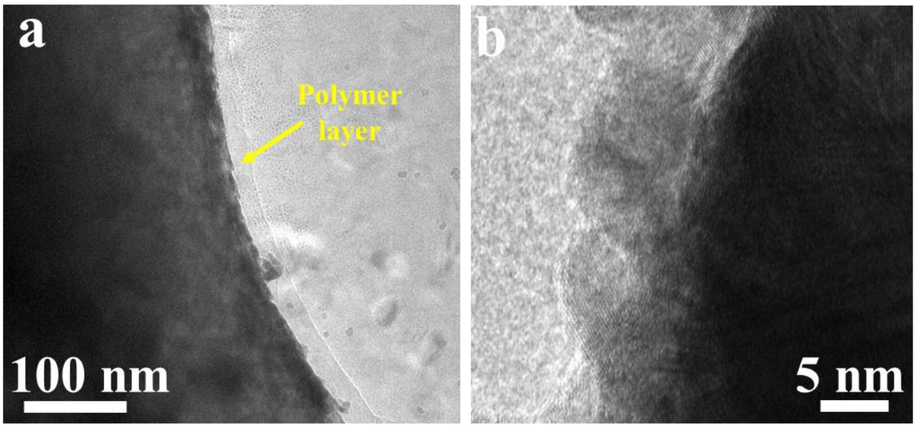

Supplementary Figure 8. a) Low-resolution Cryo-TEM image of PHS-Cu in Li||PHS-Cu after plating for 6 mAh/cm^2 at 1 mA/cm^2, 25 ^{o}C. b) Cryo-TEM image of Fig. 2a without labels.

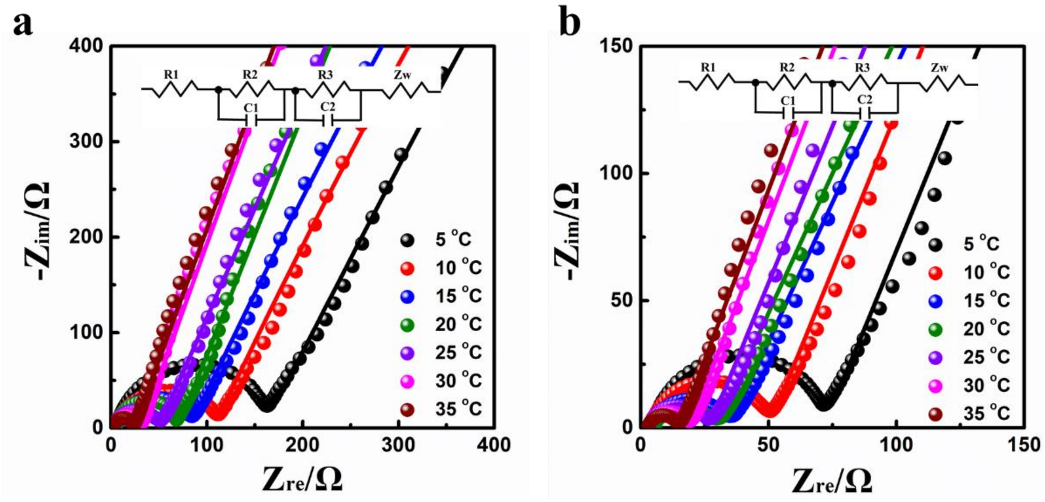

Supplementary Figure 9. Nyquist plots at various temperature form 5-35 °C for a) Li||PHS-Cu and b) Li||Cu asymmetric cells, where the symbols indicate the raw data and lines refer to fitted data, with the Chi-Squared distribution (ChiSq) between 10^{-3} and 10^{-4}. Corresponding values of R_{SEI} under various temperatures for Li||PHS-Cu and Li||Cu asymmetric cells are shown in Supplementary Table 2-3.

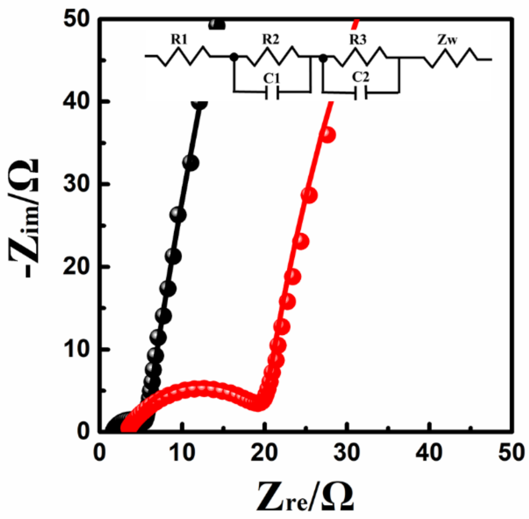

Supplementary Figure 10. Nyquist plots for Li||PHS-Cu and Li||Cu tested under 6 mAh/cm^2 with 1 mA/cm^2 at 25 ^oC after1st cycle, where the symbols indicate the raw data and lines refer to fitted data, with the Chi-Squared distribution (ChiSq) of 10^{-4}.

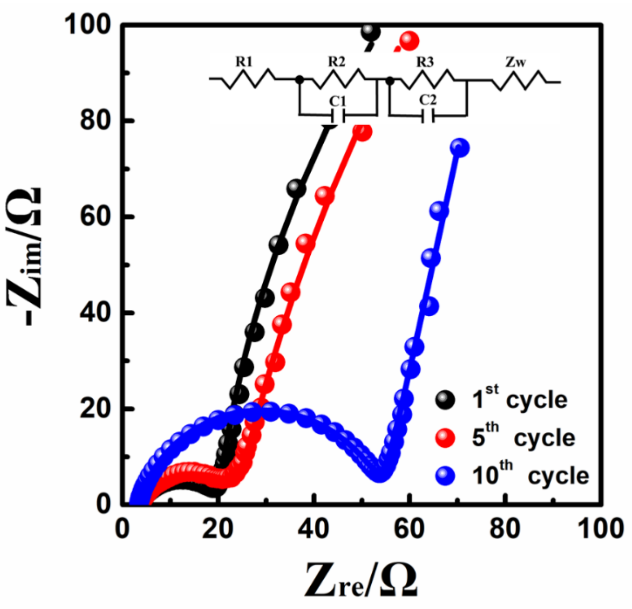

Supplementary Figure 11. Nyquist plots for Li||Cu tested at different cycles under 6 mAh/cm^2 with 1 mA/cm^2 at 25 ^{o}C, where the symbols indicate the raw data and lines refer to fitted data, with the Chi-Squared distribution (ChiSq) of 10^{-4}. Inset is the corresponding equivalent circuit.

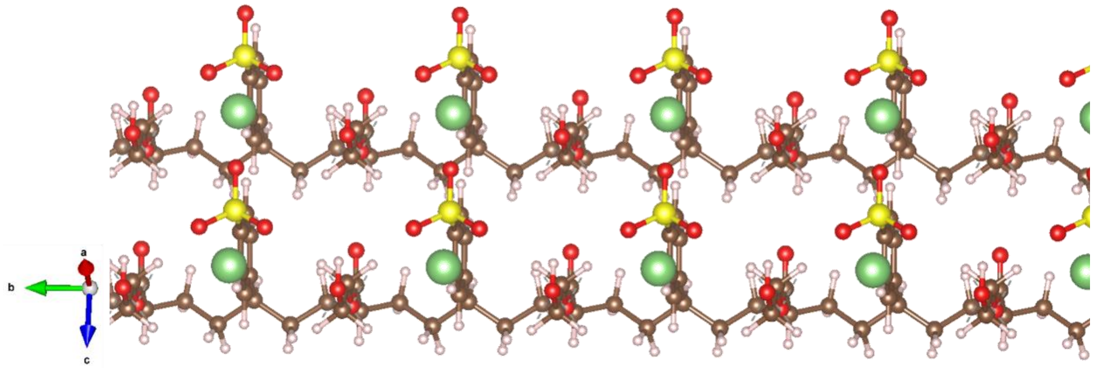

Supplementary Figure 12. Atom model for interchain migration at the initial state from side view. The green, yellow, red, brown and pink spheres indicate Li, S, O, C and H atoms, respectively.

Computational details: Quantum mechanical calculations were performed to employ three choices of exchange-correlation density functional: the PBE functional together with a Hubbard correction (abbreviated PBE+U)[1], the Perdew-Burke-Ernzerhof (PBE)[2] generalized gradient approximation, and the Heyd-Scuseria-Ernzerhof [3] screened hybrid approximation (abbreviated HSE06). It is well known that the PBE functional underestimates band gaps, and the other two functionals correct this in two different ways. The climbing-image nudged elastic band (CI-NEB)[4] method was used to determine the energy barriers for Li-ion diffusion. Five images were employed between two end points.

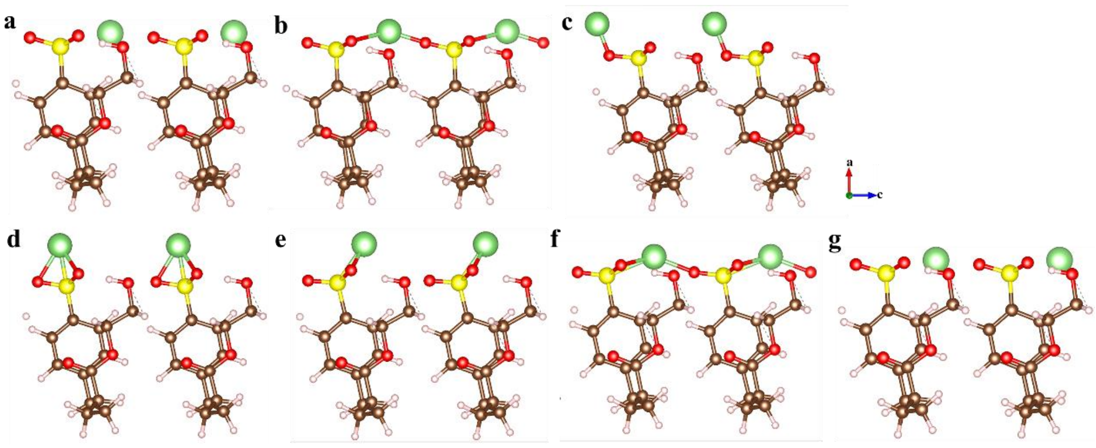

Supplementary Figure 13. Li^{+} migration in the interchain pathway from a) initial, b-f) intermediate and g) final states. The green, yellow, red, brown and pink spheres indicate Li, S, O, C and H atoms, respectively.

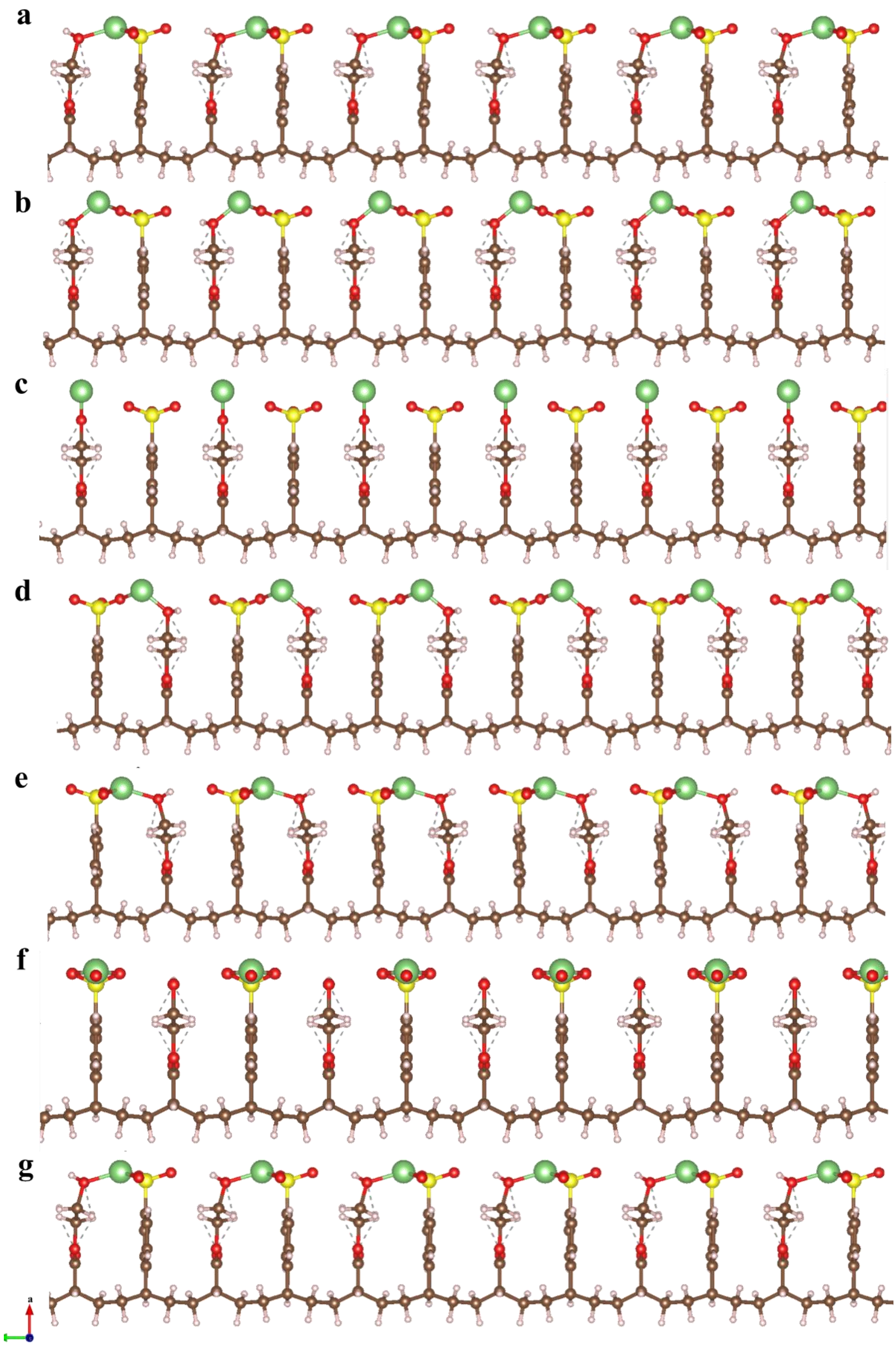

Supplementary Figure 14. Li^+ migration in the intrachain pathway from a) initial, b-f) intermediate and g) final states. The green, yellow, red, brown and pink spheres indicate Li, S, O, C and H atoms, respectively.

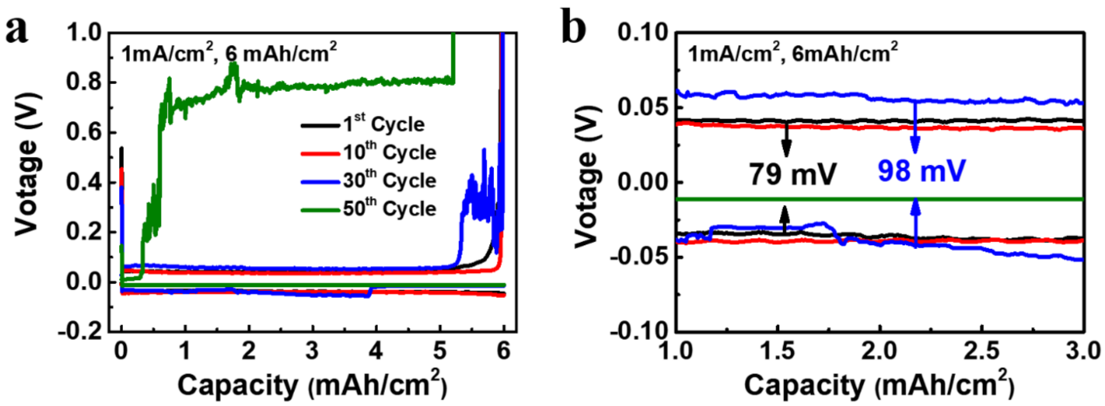

Supplementary Figure 15. a) Voltage profiles of Li||Cu asymmetric cell with 1 mA/cm^2 under 6 mAh/cm^2 at 25 ^{o}C. b) is the enlarged Supplementary Fig. 15a.

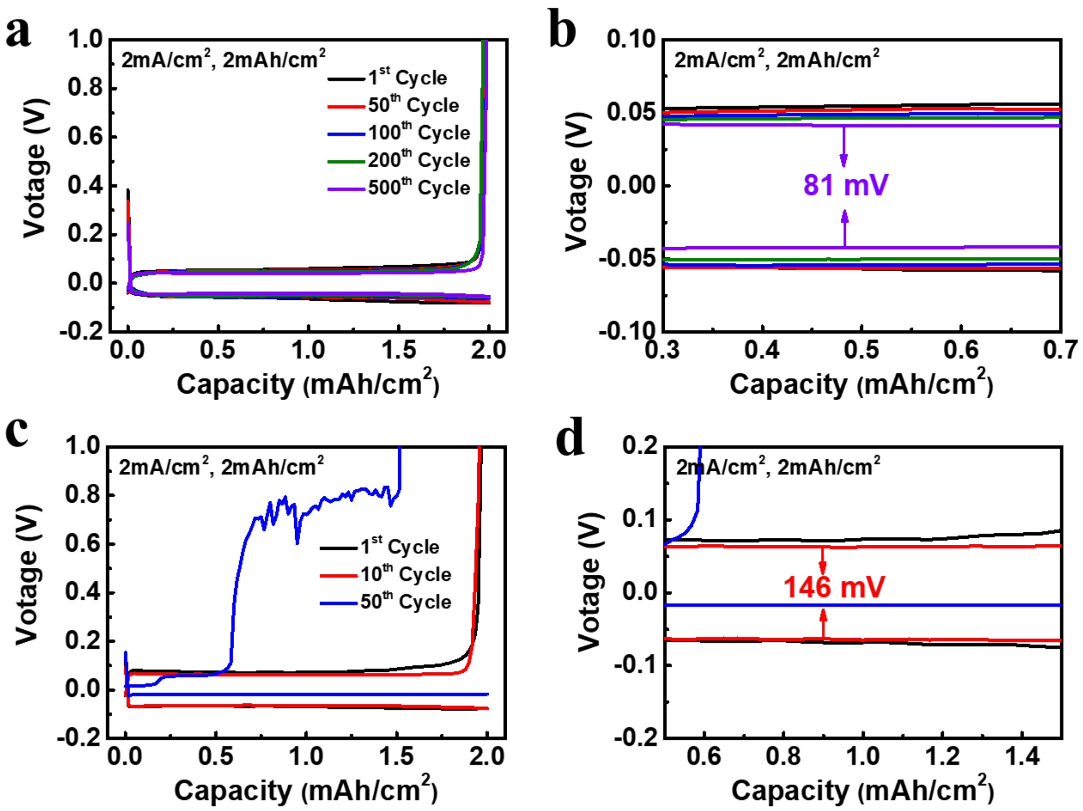

Supplementary Figure 16. Voltage profiles of a-b) Li||PHS-Cu and c-d) Li||Cu asymmetric cell with 2 mA/cm^2 under 2 mAh/cm^2 at 25 ^{o}C.

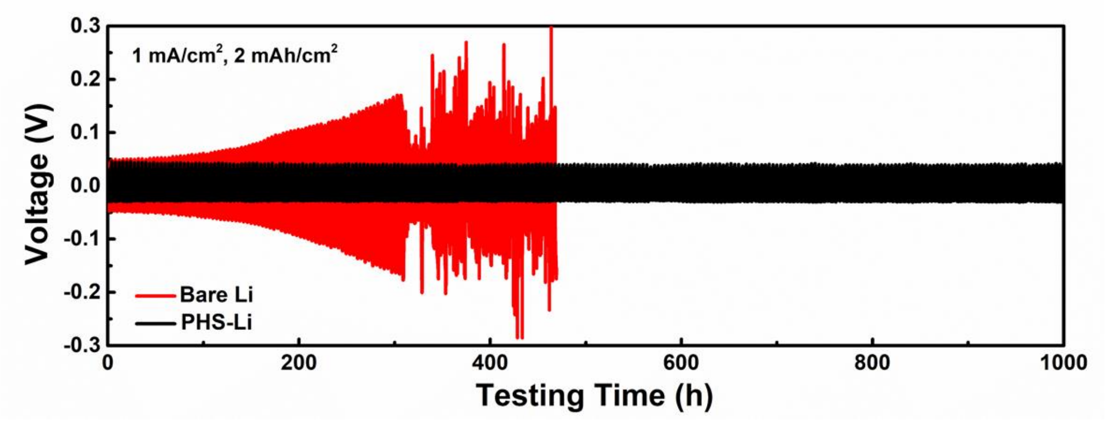

Supplementary Figure 17. Electrochemical performance of PHS-Li||PHS-Li and Li||Li symmetric cells tested at 25 ^oC.

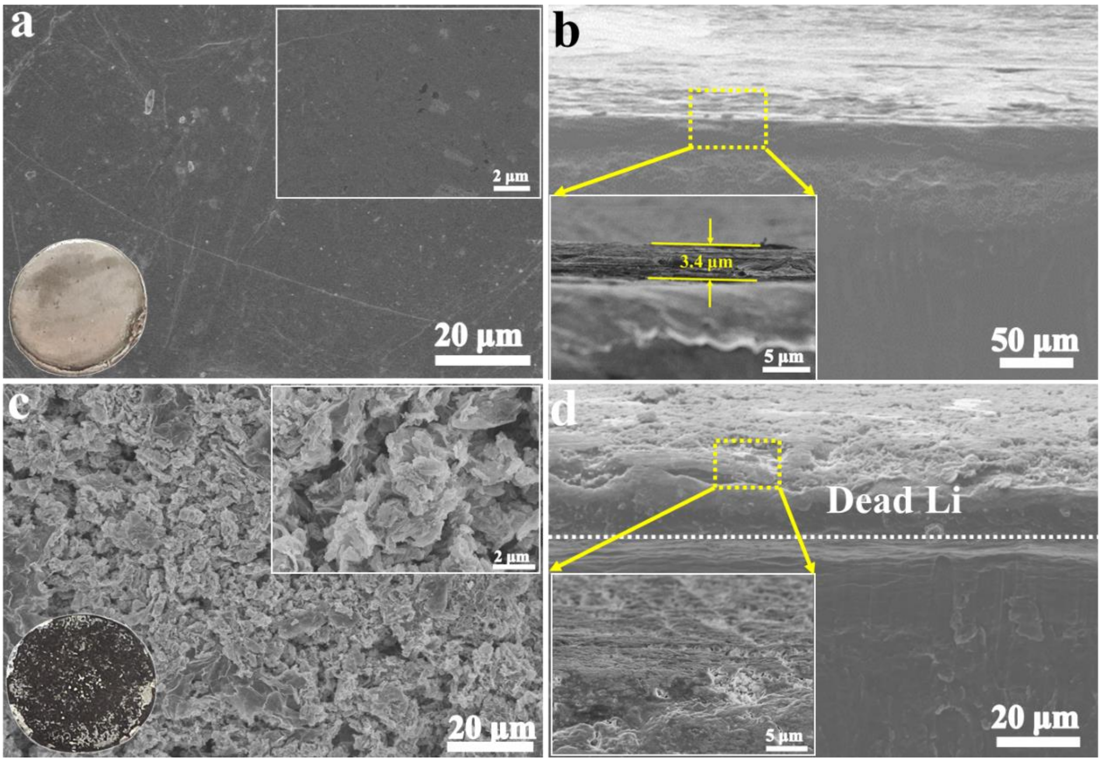

Supplementary Figure 18. a, c) SEM images and b, d) Cross-sectional SEM images of a-b) PHS-Li||PHS-Li and c-d) Li||Li after cycling for 50 cycles at 1 mA/cm^2 with 2 mAh/cm^2 at 25 ^{o}C.

In the SEM images, we observe that the smooth and flat surface can be obtained for PHS-Li without dead lithium formation, while the bare Li is covered with dead Li, indicating that PHS coated on Li can benefit for uniform deposition of Li, in accordance with coating on Cu. Notably, the thin layer on the surface of Li should be the PHS layer, implying that PHS is stable without destruction during cycling (inset of Supplementary Fig.18b).

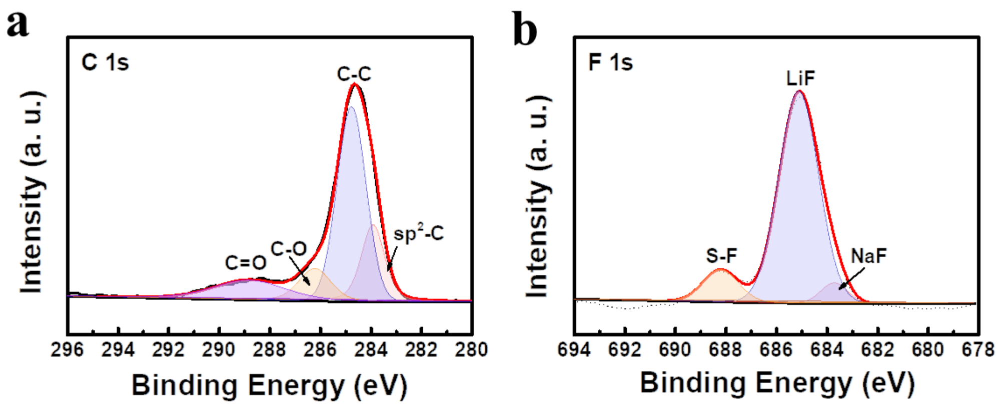

Supplementary Figure 19. XPS spectra of a) C 1*s* and b) F 1*s* for PHS-Li in PHS-Li||PHS-Li after 50th cycles with 2 mAh/cm^2 at 1 mA/cm^2, 25 ^{o}C. The C 1*s* and F 1*s* are almost the same as PHS-Cu after cycling (Fig. 5k), suggesting that PHS can uniform the Li deposition and stabilize the SEI.

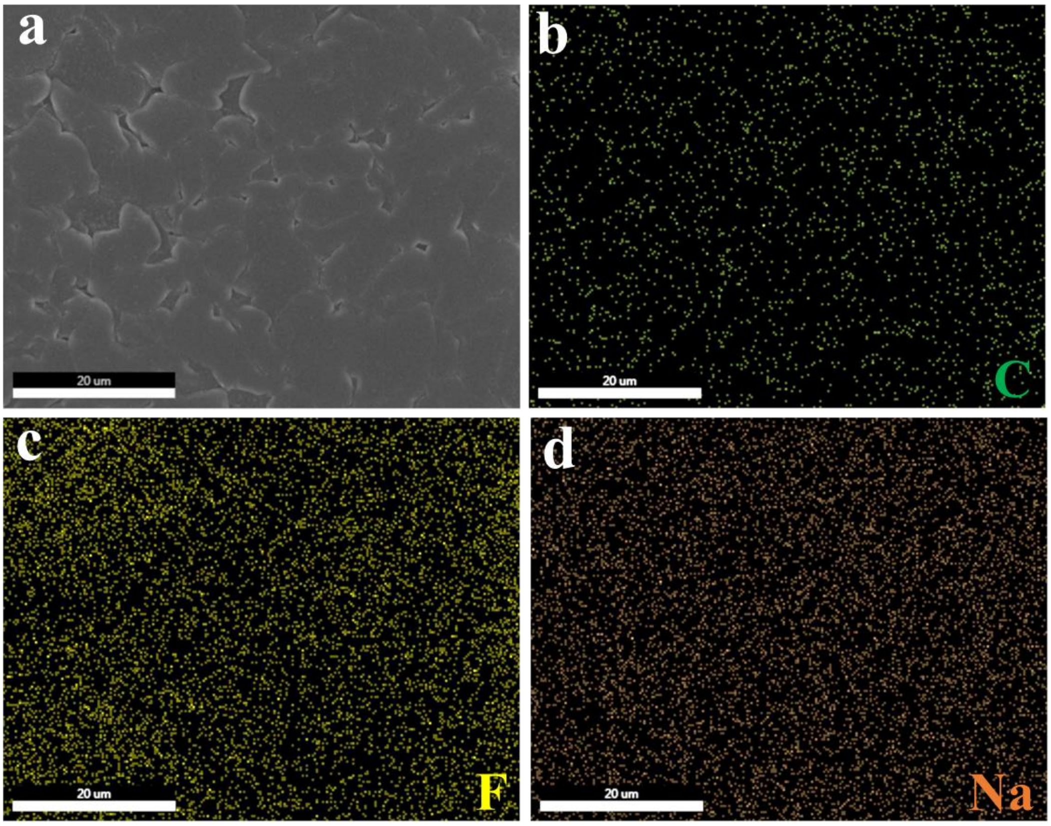

Supplementary Figure 20. EDS elemental mapping of PHS-Cu after 20 cycles under 6 mAh/cm^2 at 1 mA/cm^2, 25 ^{o}C. a) SEM image, b-d) The elemental distribution of C, F, Na.

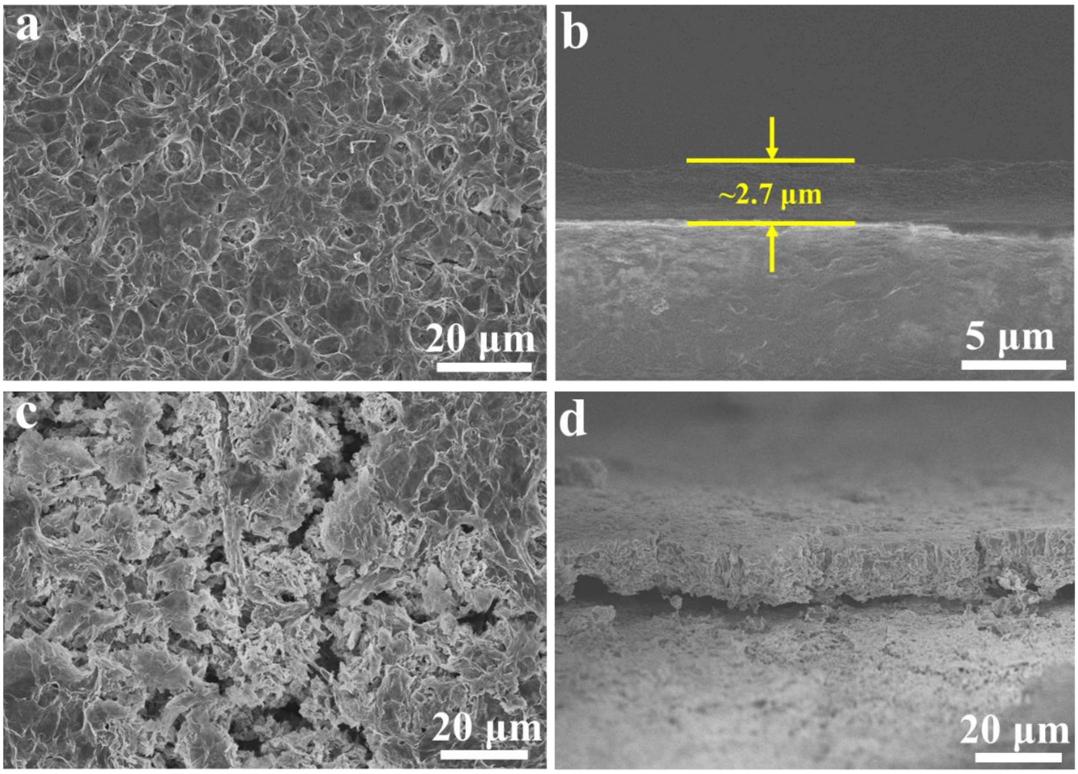

Supplementary Figure 21. SEM images of a-b) PHS-Cu in Li||PHS-Cu and c-d) Bare Cu in Li||Cu after plating/stripping with 6 mAh/cm^2 at 1 mA/cm^2, 25 ^{o}C for 1st cycle.

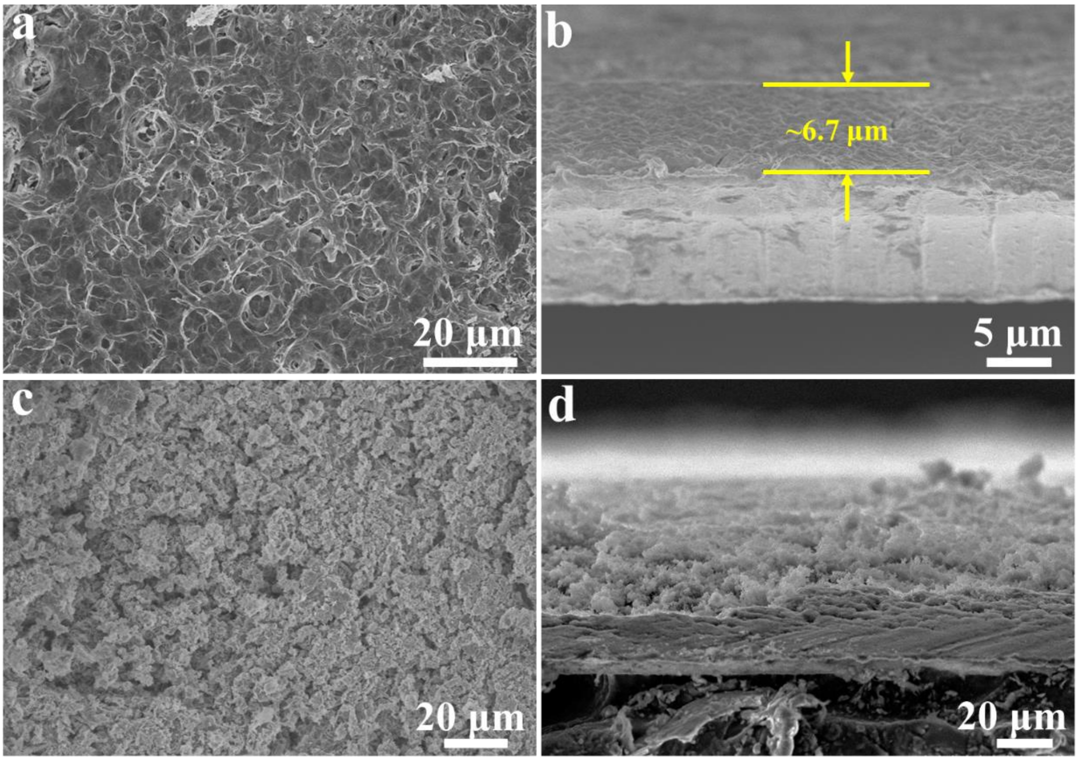

Supplementary Figure 22. SEM images of a-b) PHS-Cu in Li||PHS-Cu and c-d) Bare Cu in Li||Cu after plating/stripping with 6 mAh/cm^2 at 1 mA/cm^2, 25 ^{o}C for 20 cycles.

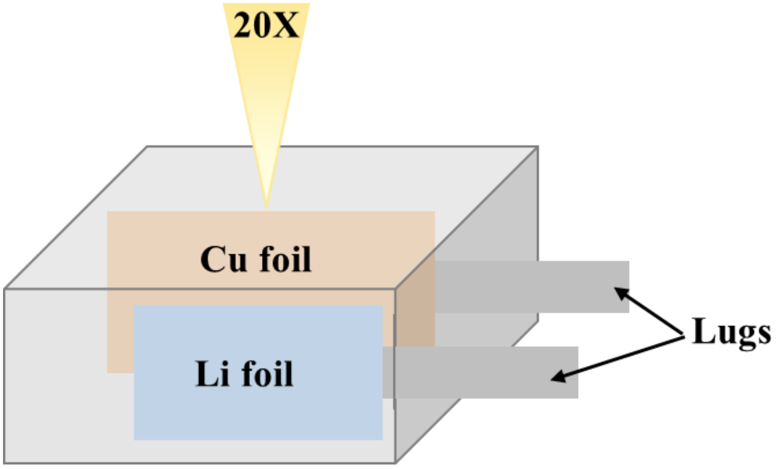

Supplementary Figure 23. Schematic diagram of asymmetric cell assembled in the homemade glassware for in situ optical microscopy test.

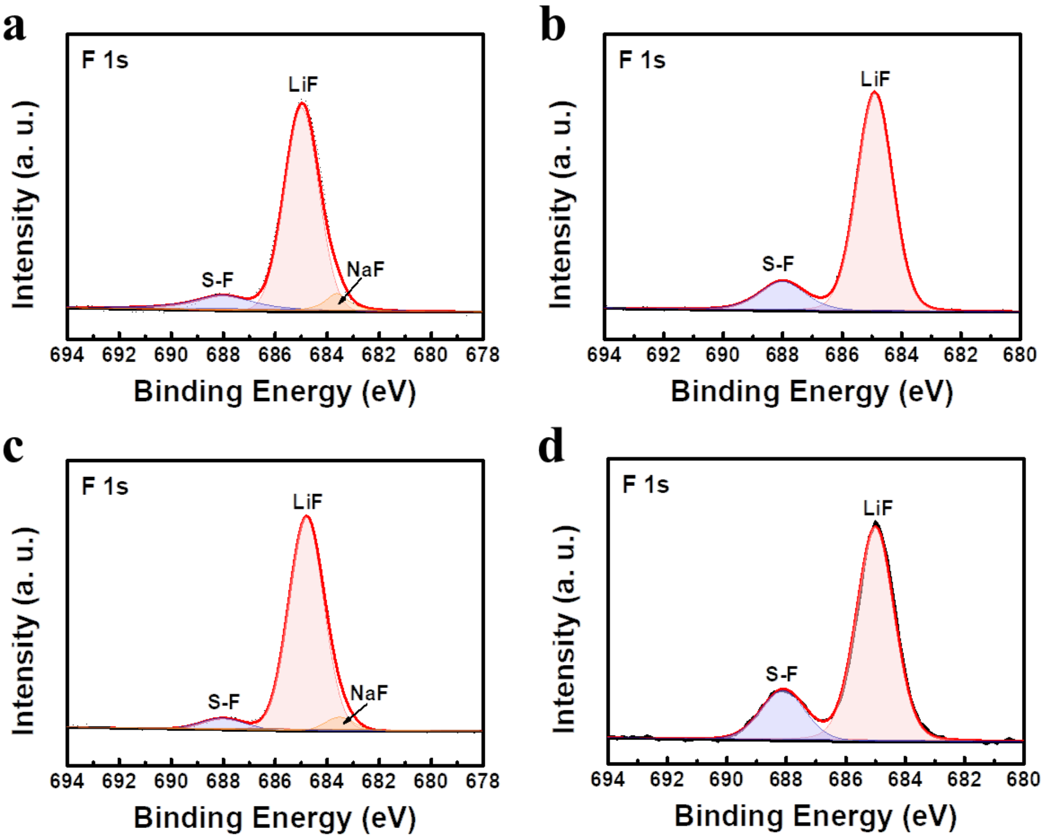

Supplementary Figure 24. XPS spectra of F 1*s* for a,c) PHS-Cu in Li||PHS-Cu and b,d) bare Cu in Li||Cu after a,b) 1st cycle and c,d) 20 cycles under 6 mAh/cm^2 at 1 mA/cm^2, 25 ^{o}C.

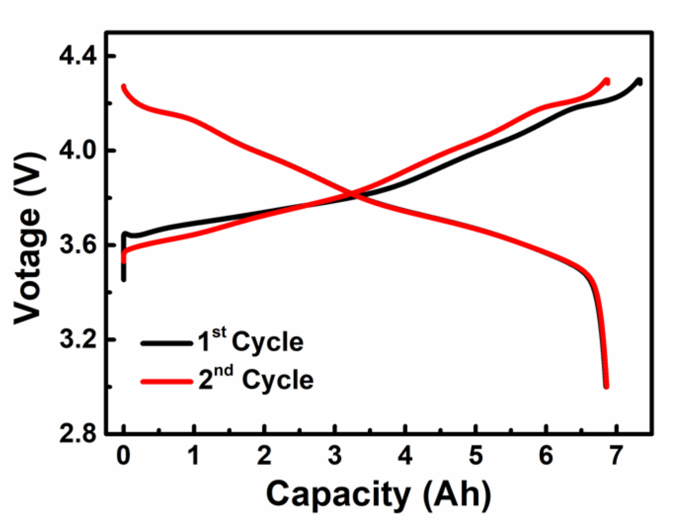

Supplementary Figure 25. The voltage profiles of PHS-Li||NCM83 pouch cell during battery formation process at 25 °C and 172 kPa.

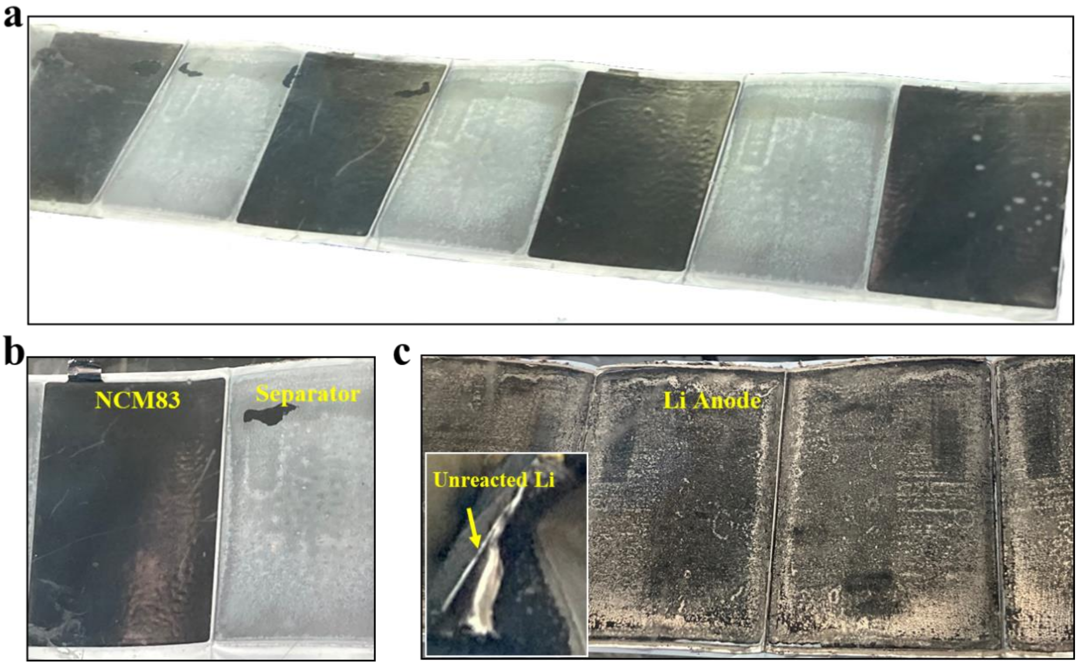

Supplementary Figure 26. Postmortem optical photographs of PHS-Li||NCM83 pouch cell after cycling for 150 cycles at 0.5 mA/cm^2 for charging and 2.5 mA/cm^2 for discharging at 25 ^{o}C. a) Disassembled pouch cell. b) Enlarged cathode. c) PHS-Li anode. Inset is the cross-sectional photograph of anode. The sizes of the positive and negative electrodes are 5.6×8.0 cm and 5.8×8.2 cm, respectively.

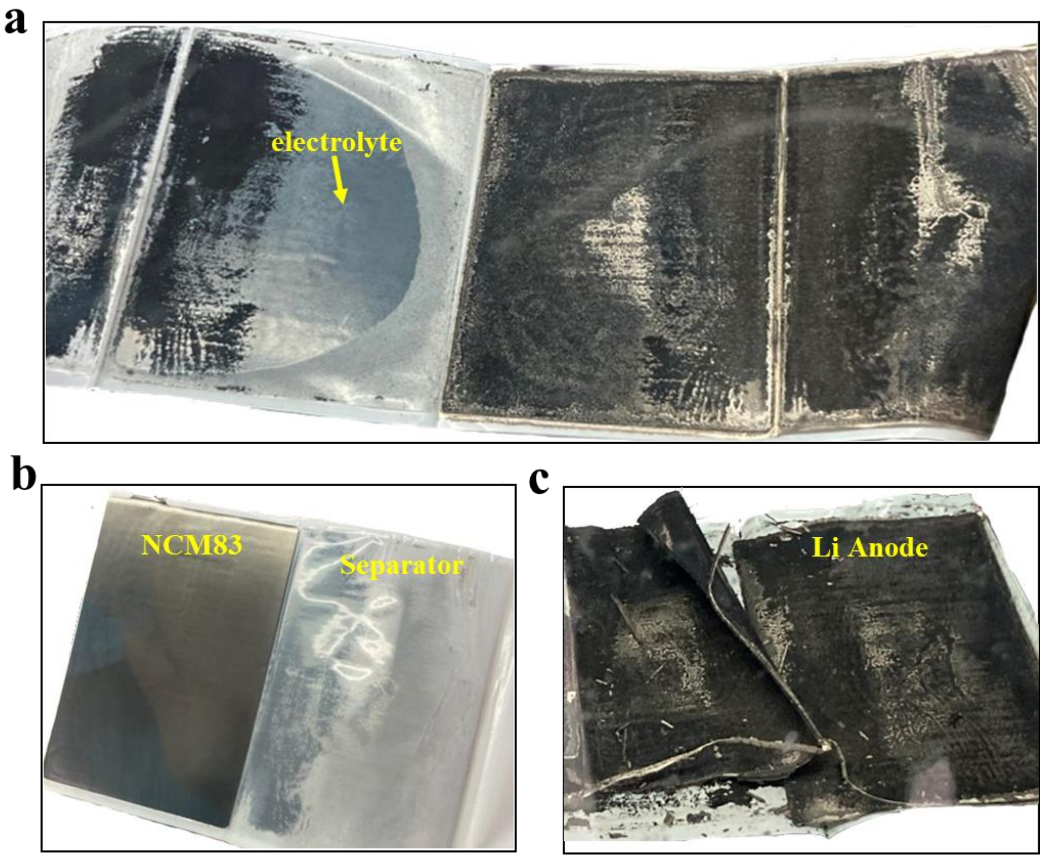

Supplementary Figure 27. Postmortem optical photographs of Li||NCM83 pouch cell after cycling for 42 cycles at 0.5 mA/cm^2 for charging and 2.5 mA/cm^2 for discharging at 25 ^oC. a) Disassembled pouch cell. b) NCM83 cathode. c) Li anode. The sizes of the positive and negative electrodes are 5.6×8.0 cm and 5.8×8.2 cm, respectively.

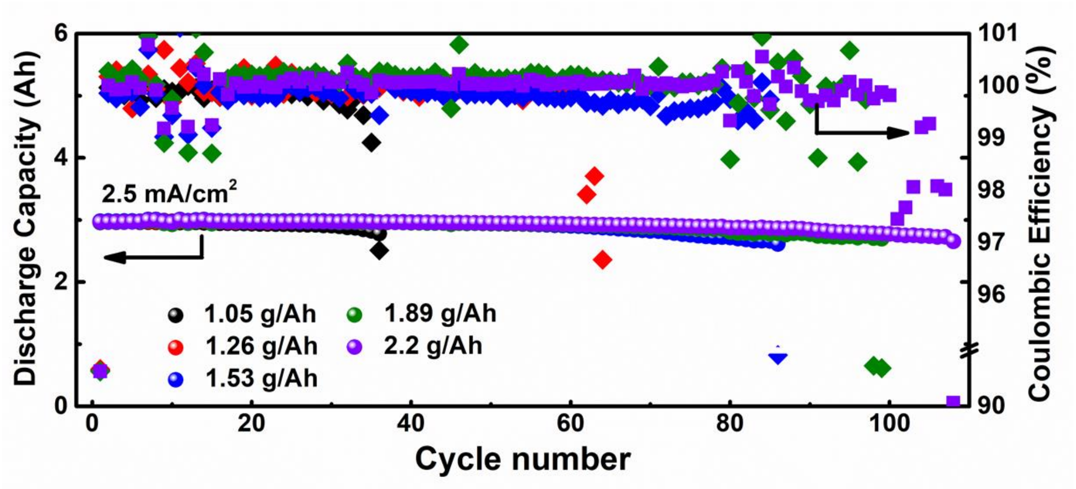

Supplementary Figure 28. Cycling performance of Li||NCM83 pouch cells with different electrolyte amounts tested at 0.5 mA/cm^2 for charging and 2.5 mA/cm^2 for discharging at 25 °C and 172 kPa. To obtain the different electrolyte amount, the electrolyte is extracted for 6s under the various vacuum degrees of -99.5, -98.5, -97, -94 and -90 kPa, where the pouch cells with the electrolyte amounts of 1.05, 1.26, 1.53, 1.89 and 2.2 g/Ah are assembled, respectively.

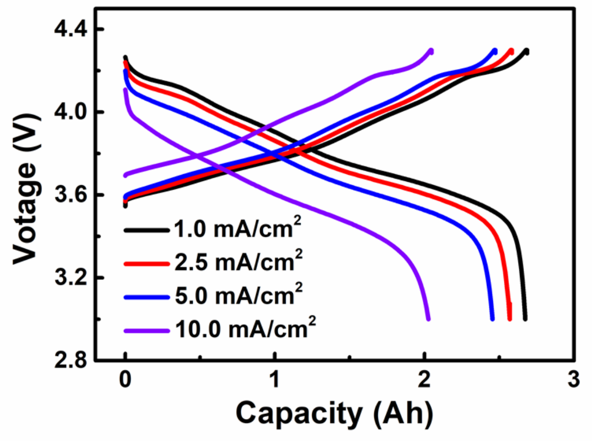

Supplementary Figure 29. The voltage profiles of PHS-Li||NCM83 pouch cell at various discharging current densities of 1, 2.5, 5 and 10 mA/cm^2 with the constant charging current density of 0.5 mA/cm^2 at 25 °C and 172 kPa.

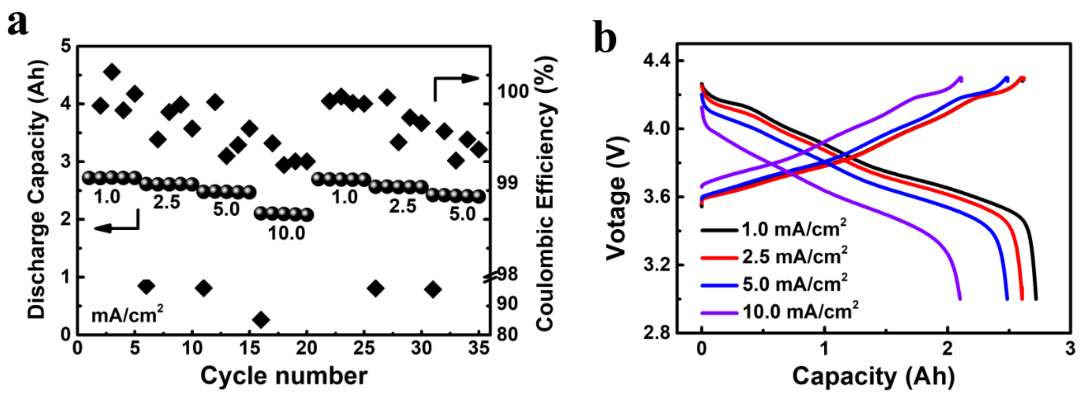

Supplementary Figure 30. a) Rate performance of PHS-Li||NCM83 pouch cell at various discharging current densities of 1, 2.5, 5 and 10 mA/cm^2 with the constant charging current density of 1 mA/cm^2 at 25 °C and 172 kPa. b) The corresponding voltage profiles.

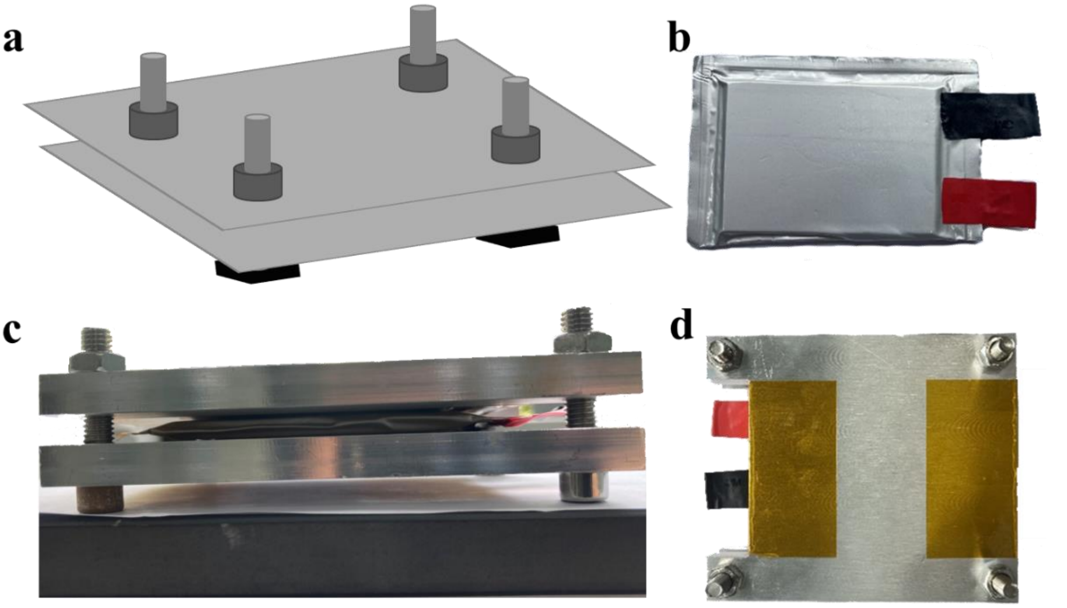

Supplementary Figure 31. a) The schematic diagram of stainless-steel clamping device. The optical photographs of b) pouch cell (7×11×0.51cm (w×l×h)), pouch cell sandwiched in clamping device from c) front view and d) top view. The size of the clamping device is 12×14×1cm (w×l×h).

Electrolyte amount (g/Ah) \ Areal capacity (mAh/cm^2)	4	5	6
3.0	376.41 Wh/kg	392.48 Wh/kg	400.06 Wh/kg
2.5	396.03 Wh/kg	413.85 Wh/kg	422.28 Wh/kg
2.0	417.8 Wh/kg	437.68 Wh/kg	447.13 Wh/kg
1.5	442.1 Wh/kg	464.43 Wh/kg	475.08 Wh/kg
1.0	469.41 Wh/kg	494.66 Wh/kg	506.76 Wh/kg
0.5	500.31 Wh/kg	529.09 Wh/kg	542.96 Wh/kg

Supplementary Table 1. The electrolyte amount and the corresponding specific energy with different cathode areal capacities of 4, 5, 6 mAh/cm^2. The Li||NCM83 pouch cell parameters are kept the same as that in Supplementary Table 3 except for electrolyte amount and cathode loading. The N/P ratio is 2.5.

T(K)	1000/T(1/K)	R_{SEI} (Ω)	Ln(T/R_{SEI})
278	3.59	67.42	1.41
283	3.53	46.27	1.81
288	3.47	30.45	2.25
293	3.41	24.66	2.47
298	3.36	21.32	2.63
303	3.3	17.12	2.87
308	3.26	12.8	3.18

Supplementary Table 2. The corresponding values of R_{SEI} under various temperatures in Kelvin for Li||PHS-Cu in Supplementary Fig. 9a.

T(K)	1000/T(1/K)	R_{SEI} (Ω)	Ln(T/R_{SEI})
278	3.59	155	0.58
283	3.53	103.4	1.01
288	3.47	70.08	1.40
293	3.41	65.27	1.50
298	3.36	47.26	1.84
303	3.3	30.68	2.29
308	3.26	20.5	2.71

Supplementary Table 3. The corresponding values of R_{SEI} under various temperatures in Kelvin for Li||Cu in Supplementary Fig. 9b.

Electrode coating strategy	Areal capacity (mAh/ cm^2)	Electrolyte	Current (mA/cm^2)	Average CE	Cycles	Tempe -rature	Ref.
Reactive polymer composite derived SEI	3	1 M LiTFSI+ $LiNO_3$ (4wt%) in DOL/DME (1:1 v/v)	2	98.5%	220	25 ºC	Ref. 5
Dynamic single-ion-conductive Artificial SEI	1	1 M $LiPF_6$ in EC/DEC	1	94.9%	250	25 ºC	Ref. 6
Biomacromolecules modified Li anode	1	1 M LiTFSI in DOL/DME (1:1 v/v)	1	98%	200	25 ºC	Ref. 7
Fluorinated mesocarbon anode	4.8	1 M LiFSI in LiDFOB-FEC	1	98.5%	150	25 ºC	Ref. 8
Electrochemically active monolayer	6	1 M $LiPF_6$ in PC/FEC (8:1 v/v)	6	98.6%	250	-15 ºC	Ref. 9
Gradient inorganic-organic coating on Lithium	0.5	1 M LiFSI in EC/DEC/DMC (1:1:1 v/v/v)	1	98%	350	25 ºC	Ref. 10
SnI_4 modified Li anode	3	1 M LiFSI in FEC/DMC (1:4 v/v)	3	98.7%	120	25 ºC	Ref. 11
TMSB-modified electrolyte	1	1.5 M LiFSI/ TMSB (3wt%) in FEC (34.7 wt%)/EMC(57.2 wt%)/DMC(8.2 wt%)	1	97.94%	500	25 ºC	Ref. 12
Au@ZIF-8	1	1 M LiTFSI+0.2 M $LiNO_3$ in DOL/DME (1:1 v/v)	10	98%	250	25 ºC	Ref. 13
lithium-montmorillonite SEI	3	LS-002	1	98.2	60	25 ºC	Ref. 14
Epitaxial induced plating Cu	5	6 M LiFSI in DME	0.5	99.24%	30	25 ºC	Ref. 15
Metal fluoride spansules	1	1 M LiTFSI in DOL/DME (1:1 v/v)	5	98.5%	300	25 ºC	Ref. 16
Protein molecules modified electrolyte	1	1 M LiTFSI in DOL/DME (1:1 v/v)	1	98%	100	25 ºC	Ref. 17
Vertically aligned GO anode	5	1 M LiTFSI+1% $LiNO_3$ in DOL/DME (1:1 v/v)	5	99.08%	150	25 ºC	Ref. 18
PHS modified Cu anode	2	4 M LiFSI in DME	1	99.45%	600	25 ºC	This work
	6	4 M LiFSI in DME	1	99.46%	150	25 ºC	
	8	4 M LiFSI in DME	1	99.43%	100	25 ºC	

Supplementary Table 4. Summary of literature research work on Li||Cu cell testing.

Cell component	Specification	Parameters
Cathode(NCM83)	Number of layers	13
	Active material mass loading (mg/cm^2, each side)	30.0
	Active material content	0.955
	Discharge capacity (mAh/g)	210
	Specific areal capacity (mAh/cm^2, each side)	6.02
	NCM Mass (g)	34.944
	Thickness (μm) of Al foil	12
	Thickness (μm) of cathode	172
	The Mass of Al foil（g）	1.863
	The Mass of Al foil + NCM (g)	36.81
Anode Li	Number of layers	14
	Thickness (μm)	100 (50 μm for each side)
	Mass (g)	3.95
Electrolyte solution	electrolyte/capacity (g/Ah)	1.25
	Mass (g)	8.58
Separator	Mass (g)	1.89
package and lugs	Mass (g)	2.0
	Dimension (cm×cm)	7x11
Full cell	Discharge capacity (Ah)	6.86
	Average discharge voltage (V)	3.8
	Total Mass (g)	53.23
	Energy density (Wh/L)	752.08
	Specific energy (Wh/kg)	489.7

Supplementary Table 5. The specifications of the PHS-Li||NCM83 pouch cell tested at 0.5 mA/cm^2 for charging and 2.5 mA/cm^2 for discharging at 25 ^oC. Notably, total mass of the full cell is obtained by directly weighing the pouch cell on analytical balance. The electrolyte amount is estimated by the total mass of pouch cell and the other parts because the pouch cell undergoes secondary electrolyte extraction (extraction for 6 seconds under the vacuum degree of -98.5 kPa). Owing to the thin layer and low density of PHS, the mass of PHS layer can be negligible and included in the electrolyte dosage.

Cell component	Specification	Parameters (Li-1#)	Parameters (Li-2#)	Parameters (Li-3#)	Parameters (Li-4#)	Parameters (Li-5#)
Cathode (NCM83)	Number of layers	6	6	6	6	6
	Active material mass loading (mg/cm^2, each side)	30	30	30	30	30
	Active material content	0.955	0.955	0.955	0.955	0.955
	Discharge capacity (mAh/g)	210	210	210	210	210
	Specific areal capacity (mAh/cm^2, each side)	6.02	6.02	6.02	6.02	6.02
	NCM Mass (g)	16.128	16.128	16.128	16.128	16.128
	Thickness (μm) of Al foil	12	12	12	12	12
	Thickness (μm) of cathode	172	172	172	172	172
	The Mass of Al foil（g)	0.86	0.86	0.86	0.86	0.86
	The Mass of Al foil + NCM (g)	16.988	16.988	16.988	16.988	16.988
Anode Li	Number of layers	7	7	7	7	7
	Thickness (μm)	100 (50 μm for each side)	100 (50 μm for each side)	100 (50 μm for each side)	100 (50 μm for each side)	100 (50 μm for each side)
	Mass (g)	1.975	1.975	1.975	1.975	1.975
Electrolyte solution	electrolyte/capacity (g/Ah)	1.05	1.26	1.53	1.89	2.2
	Mass (g)	3.11	3.73	4.55	5.59	6.52
Separator	Mass (g)	0.91	0.91	0.91	0.91	0.91
package and lugs	Mass (g)	1.96	1.96	1.96	1.96	1.96
	Dimension (cm×cm)	7x11	7x11	7x11	7x11	7x11
Full cell	Discharge capacity (Ah)	2.966	2.964	2.977	2.960	2.965
	Average discharge voltage (V)	3.8	3.8	3.8	3.8	3.8
	Total Mass (g)	24.943	25.563	26.383	27.423	28.353
	Specific energy (Wh/kg)	451.9	440.6	428.8	410.2	397.4

Supplementary Table 6. The specifications of the Li||NCM83 pouch cells with different electrolyte amounts tested at 0.5 mA/cm^2 for charging and 2.5 mA/cm^2 for discharging at 25 °C. The total mass of the full cell is obtained by directly weighing the pouch cell on analytical balance. The electrolyte amount is estimated by the total mass of pouch cell and the other parts because the pouch cell undergoes secondary electrolyte extraction.

Cell component	Specification	Parameters
Cathode(NCM83)	Number of layers	5
	Active material mass loading (mg/cm^2, each side)	30.0
	Active material content	0.955
	Discharge capacity (mAh/g)	210
	Specific areal capacity (mAh/cm^2, each side)	6.02
	NCM Mass (g)	13.44
	Thickness (μm) of Al foil	12
	Thickness (μm) of cathode	172
	The Mass of Al foil（g）	0.716
	The Mass of Al foil + NCM (g)	14.166
Anode Li	Number of layers	6
	Thickness (μm)	100 (50 μm for each side)
	Mass (g)	1.69
Electrolyte solution	electrolyte/capacity (g/Ah)	1.28
	Mass (g)	3.52
Separator	Mass (g)	1.89
package and lugs	Mass (g)	1.93
	Dimension (cm×cm)	7x11
Full cell	Discharge capacity (Ah)	2.75
	Average discharge voltage (V)	3.8
	Total Mass (g)	23.196
	Specific energy (Wh/kg)	450.5

Supplementary Table 7. The specifications of the PHS-Li||NCM83 pouch cells at 0.5 mA/cm^2 for charging and 5 mA/cm^2 for discharging at 25 ^oC. The total mass of the full cell is obtained by directly weighing the pouch cell on analytical balance. The electrolyte amount is estimated by the total mass of pouch cell and the other parts because the pouch cell undergoes secondary electrolyte extraction.

Supplementary References

1. Dudarev S, Botton GA, Savrasov SY, Humphreys C, Sutton AP. Electron-Energy-Loss Spectra and the Structural Stability of Nickel Oxide: An LSDA+U Study. *Phys Rev B* **57**, 1505-1509 (1998).
2. Perdew JP, Burke K, Ernzerhof M. Generalized Gradient Approximation Made Simple. *Phys Rev Lett* **77**, 3865-3868 (1996).
3. Krukau AV, Vydrov OA, Izmaylov AF, Scuseria GE. Influence of the exchange screening parameter on the performance of screened hybrid functionals. *J Chem Phys* **125**, 224106 (2006).
4. Henkelman G, Uberuaga BP, Jónsson H. A climbing image nudged elastic band method for finding saddle points and minimum energy paths. *J Chem Phys* **113**, 9901-9904 (2000).
5. Zhao Y, Wang D, Gao Y, Chen T, Huang Q, Wang D. Stable Li metal anode by a polyvinyl alcohol protection layer via modifying solid-electrolyte interphase layer. *Nano Energy* **64**, 103893 (2019).
6. Yu Z, *et al.* A Dynamic, Electrolyte-Blocking, and Single-Ion-Conductive Network for Stable Lithium-Metal Anodes. *Joule* **3**, 2761-2776 (2019).
7. Ju Z, *et al.* Biomacromolecules enabled dendrite-free lithium metal battery and its origin revealed by cryo-electron microscopy. *Nat Commun* **11**, 488 (2020).
8. Cui C, *et al.* A Highly Reversible, Dendrite-Free Lithium Metal Anode Enabled by a Lithium-Fluoride-Enriched Interphase. *Adv Mater* **32**, e1906427 (2020).
9. Gao Y, *et al.* Low-temperature and high-rate-charging lithium metal batteries enabled by an electrochemically active monolayer-regulated interface. *Nat Energy* **5**, 534-542 (2020).
10. Sun Y, *et al.* Regulated lithium plating and stripping by a nano-scale gradient inorganic–organic coating for stable lithium metal anodes. *Energy Environ Sci* **14**, 4085-4094 (2021).
11. Jin CB, *et al.* Reclaiming Inactive Lithium with a Triiodide/Iodide Redox Couple for Practical Lithium Metal Batteries. *Angew Chem Int Ed* **60**, 22990-22995 (2021).
12. Huang K, *et al.* Regulation of SEI Formation by Anion Receptors to Achieve Ultra-Stable Lithium-Metal Batteries. *Angew Chem Int Ed* **60**, 19232-19240 (2021).
13. Huang M, Yao Z, Yang Q, Li C. Consecutive Nucleation and Confinement Modulation towards Li Plating in Seeded Capsules for Durable Li-Metal Batteries. *Angew Chem Int Ed* **60**, 14040-14050 (2021).
14. Nan Y, *et al.* Interlamellar Lithium-Ion Conductor Reformed Interface for High Performance Lithium Metal Anode. *Adv Funct Mater* **31**, (2021).
15. Lin L, Suo L, Hu Ys, Li H, Huang X, Chen L. Epitaxial Induced Plating Current-Collector Lasting Lifespan of Anode-Free Lithium Metal Battery. *Adv Energy Mater* **11**, 2003709 (2021).
16. Yuan H, *et al.* An ultrastable lithium metal anode enabled by designed metal fluoride spansules. *Sci Adv* **6**, 3112 (2020).
17. Wang T, *et al.* Immunizing lithium metal anodes against dendrite growth using protein molecules to achieve high energy batteries. *Nat Commun* **11**, 5429 (2020).
18. Chen H, *et al.* Tortuosity Effects in Lithium-Metal Host Anodes. *Joule* **4**, 938-952 (2020).
